# Matrix-bound nanovesicles as epigenetic modulators of myeloid cells

**DOI:** 10.1126/sciadv.adx9159

**Published:** 2026-02-11

**Authors:** Héctor Capella-Monsonís, Jiayang Rong, Bharadwaj Chirravuri, William D’Angelo, Hēth R. Turnquist, George Hussey, Stephen F. Badylak

**Affiliations:** ^1^ McGowan Institute for Regenerative Medicine, University of Pittsburgh, 450 Technology Drive, Pittsburgh, PA 15219, USA.; ^2^ Department of Surgery, School of Medicine, University of Pittsburgh, 200 Lothrop Street, Pittsburgh, PA 15213, USA.; ^3^ Viscus Biologics LLC, 2603 Miles Road, Cleveland, OH 44128, USA.; ^4^ Department of Immunology, School of Medicine, University of Pittsburgh, 5051 Centre Ave., Pittsburgh, PA 15213, USA.; ^5^ Department of Pathology, School of Medicine, University of Pittsburgh, 200 Lothrop Street, Pittsburgh, PA 15213, USA.; ^6^ Department of Bioengineering, University of Pittsburgh, 3700 O’Hara Street, Pittsburgh, PA 15261, USA.

## Abstract

Extracellular vesicles (EV) represent a conserved and highly efficient mechanism for cell-to-cell communication. Matrix-bound nanovesicles (MBV) are a recently identified type of EV embedded within the extracellular matrix with potent local and systemic immunomodulatory effects on myeloid cells. These effects are durable and last beyond the predicted life span of differentiated myeloid cells such as macrophages. The present study investigated MBV-directed epigenetic modification in myeloid precursors as a potential explanation for their prolonged immunomodulatory effects. Flow cytometry and ATAC sequencing studies show that MBV are internalized by myeloid cell progenitors in the bone marrow and in macrophages after terminal differentiation. This internalization is coincident with epigenetic changes that are associated with modulation of macrophage responses to inflammatory stimuli. Furthermore, MBV treatment differentially alters chromatin accessibility as a function of cell differentiation state (i.e., myeloid progenitor versus macrophage). The present study shows the epigenetic effects of MBV on myeloid cells, representing a potential avenue to exploit the therapeutic potential of biologic scaffold materials.

## Teaser

Matrix-bound nanovesicles elicit epigenetic changes in myeloid cells, modulating their response to future inflammatory events.

## INTRODUCTION

Matrix-bound nanovesicles (MBV) represent a distinct class of extracellular vesicles (EV) (*1*, *2*) that are embedded within the extracellular matrix (ECM) of all tissues tested to date (*1*, *3*). This tissue residence contrasts with other EV such as exosomes that are released freely into body fluids, such as the blood, saliva, urine, or cerebrospinal fluid (*4*, *5*). Isolated MBV have immunomodulatory properties that include mitigation of the proinflammatory immune cell phenotype, most notably in myeloid cells such as macrophages (*6*–*8*). MBV also generate a proreparative phenotype in macrophages that supports tissue repair and the restoration of function after injury (*6*, *9*). Of note, the anti-inflammatory effect occurs without systemic immunosuppression; that is, MBV do not compromise the ability to mount an adaptive immune response to specific antigens (*10*). Furthermore, MBV-mediated immunomodulatory effects persist far beyond the expected life span of innate effector cells derived from the bone marrow precursors, including macrophages (*11*–*13*), a finding that appears to translate to long-lasting systemic therapeutic effects as shown in several preclinical in vivo models (*14*–*17*). These observations may suggest a mechanism of action that involves not simply the immediate inhibition of particular inflammatory mediators in myeloid cells, such as tumor necrosis factor–α (TNF-α) or interleukin-6 (IL-6), but rather may reflect the induction of a heritable change in gene expression that could be driven by MBV-mediated epigenetic regulation in innate cell precursors. Epigenetic regulation involves the molecular transformation of DNA to modulate gene expression independent of the nucleotide sequence (*18*), resulting in inheritable regulated gene expression. The most common mechanisms of epigenetic regulation include DNA methylation and chromatin remodeling such as histone methylation or acetylation (*18*–*20*). Limited data suggest that EVs elicit epigenetic regulation to contribute to immunomodulatory effects (*21*–*24*). The potential for MBV-mediated epigenetic modifications and their contribution to functional modifications of macrophages has not been investigated.

The objective of the present study was to evaluate potential epigenetic effects of MBV upon myeloid cells, both in vitro and in vivo. The impact of MBV on myeloid progenitors and monocyte-derived macrophages at the epigenetic level was a main focus. Here, it is shown that myeloid cells, including myeloid progenitor cells, uptake MBV and undergo epigenetic alterations that can be passed to their descendant differentiated cells. These findings suggest a potential long-lasting therapeutic use of MBV and a step forward in our understanding of ECM function.

## RESULTS

### MBV accumulate in the bone marrow

**Fig. 1.**
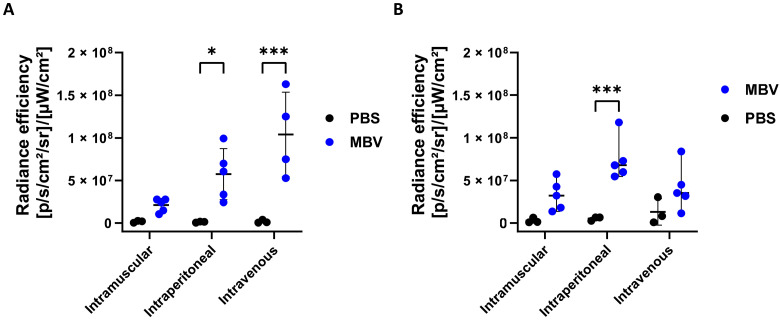
IVIS analysis that MBV injected systemically in mice reach the bone marrow. IVIS analysis demonstrated that MBV injected systemically in mice reach the bone marrow. Quantification of the average radiance efficiency showed significant signal 3 hours (**A**) after 10^12^ MBV/ml injection intramuscularly, intraperitoneally, and intravenously, while only intraperitoneal injection generated a significant signal after 24 hours (**B**) as opposed to intramuscular and intravenous delivery [* and *** indicate *P* < 0.05 and 0.001 (*N* = 5) with PBS (*N* = 3), respectively].

To test whether MBV traffic to the bone marrow after injection in mice, we injected DiD-tagged MBV intramuscularly (IM), intravenously (IV), or intraperitoneally (IP) and isolated the bone marrow 3 and 24 hours posttreatment for In Vivo Imaging System (IVIS) imaging. No signal was observed in leukocytes from blood from any group or time point (fig. S2, A and B). This indicates that MBV are rapidly lost from the circulation (within 24 hours), in agreement with a previous study (*25*). At 3 hours after injection of MBV, bone marrow from IP and IV groups (Fig. 1A) accumulated a significant signal, the highest being observed in the IV group. In all cases, the signal measured was lower than the single dose positive control of fluorescently tagged MBV (fig. S2B), which indicates that only a small fraction of the injected MBV reaches the bone marrow. By 24 hours only, the IP-delivered MBV showed a significant signal (Fig. 1B). The lower signal in the 24-hour IV group may be accounted for by rapid uptake and processing of MBV by bone marrow resident cells, while the low signal observed in the IM injection group could be due to MBV immobilization in the local injection area (*25*). These results confirm, however, that MBV accumulate in the bone marrow after IV and IP delivery, with the greatest accumulation at 24 hours in the IP group.

### Myeloid cell populations uptake MBV

**Fig. 2.**
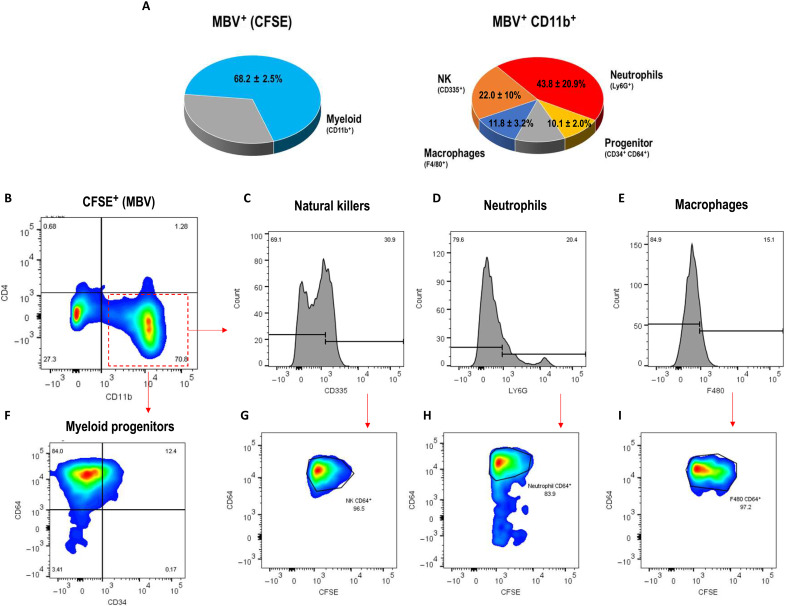
Flow cytometry analysis in bone marrow cells exposed to MBV for 3 hours in vitro. Flow cytometry of bone marrow cells after in vitro exposure to 1 × 10^11^ MBV/ml showed that 68.2% of cells interacting with MBV (CFSE^+^) after 3 hours were CD11b^+^ (**A**). CD11b^+^ cells were further classified according to markers for NK cells (CD335^+^), macrophages (F4/80^+^), neutrophils (Ly6G^+^), or myeloid progenitors (CD34^+^/CD64^+^). Percentages showed as average of *N* = 3. Gated MBV/CFSE^+^ cells showed an absence of T cell and a clear majority of CD11b^+^ cells (**B**). These CFSE^+^/CD11b^+^ cells included CD335^+^ NK cells (**C**), Ly6G^+^ neutrophils (**D**), F4/80^+^ macrophages (**E**), and CD64^+^/CD34^−^ myeloid progenitors (**F**). CFSE^+^/CD11b^+^ cells were in their majority also CD64^+^, including high percentages of NK cells (**G**), neutrophils (**H**), and macrophages (**I**).

**Fig. 3.**
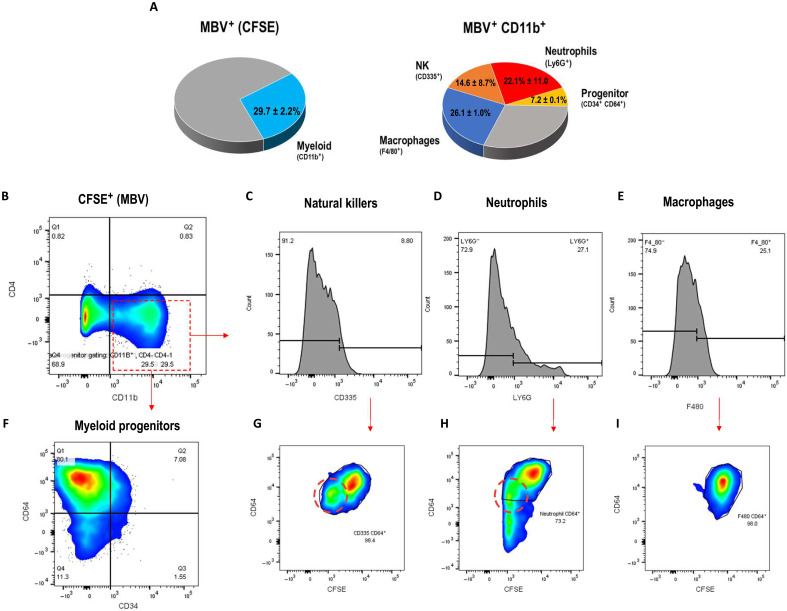
Flow cytometry analysis in bone marrow cells exposed to MBV for 24 hours in vitro. Flow cytometry of bone marrow cells after in vitro exposure to 1 × 10^11^ MBV/ml showed that 29.7% of cells interacting with MBV (CFSE^+^) after 24 hours were CD11b^+^ (**A**). CD11b^+^ cells were further classified as NK cells (CD335^+^), macrophages (F4/80^+^), neutrophils (Ly6G^+^), or myeloid progenitors (CD34^+^/CD64^+^). Percentages showed as average of *N* = 3. Gated MBV/CFSE^+^ cells showed an absence of T cell and a clear majority of CD11b^+^ cells (**B**). These CFSE^+^/CD11b^+^ cells included CD335^+^ NK cells (**C**), Ly6G^+^ neutrophils (**D**), F4/80^+^ macrophages (**E**), and CD64^+^/CD34^−^ myeloid progenitors (**F**), CFSE^+^/CD11b^+^ cells were in their majority also CD64^+^, including high percentages of natural killers (NK cells) (**G**), neutrophils (**H**), and macrophages (**I**). Both NK cells and neutrophils showed subpopulations of CD64^dim^ (red dashed circles).

While accumulation of DiD-labeled MBV was observed in the bone marrow following in vivo administration of 10^11^ MBV, carboxyfluorescein diacetate succinimidyl ester (CFSE) signal was too low to enable flow cytometric detection and characterization of the MBV-interacting cells in vivo. Thus, we treated bone marrow cells ex vivo with CFSE-labeled MBV for 3 or 24 hours and identified CFSE^+^ cells using flow cytometry (representative staining and gating showed in fig. S3). After 3 hours, less than 0.5% of cells showed MBV uptake, while at 24 hours, this percentage increased to ~1.5% (fig. S3, A and D). Of the cells that interacted with MBV, almost 70% were myeloid cells (CD11b^+^) at 3 hours (Fig. 2A), while this proportion decreased by ~30% after 24 hours (Fig. 3A), which indicates that cells of other lineages can actively take up MBV as well, as previously reported (*25*). This earlier uptake of MBV by CD11b^+^ cells compared to other cell types could be explained by their phagocytic capability. Among myeloid cells that internalized MBV, the highest proportion was identified as neutrophils, followed by natural killer cells (NK cells), and macrophages. Approximately 7 to 10% of these cells were CD11b^+^ CD34^+^ CD64^+^ (Figs. 2A and 3A), which are recognized as monocytic-committed progenitor cells that differentiate into the various granulocyte and myeloid subtypes (*26*, *27*). Considering that progenitor cells have a lower phagocytic capacity than terminally differentiated myeloid cells (*28*), the current results suggest a different mechanism by which MBV interact with progenitor cells.

The proportion of CD4^+^ T cells that took up MBV was negligible. Within the MBV^+^ CD11b^+^ population, 30.9% were CD335^+^ NK cells, 20.4% were Ly6G^+^ neutrophils, 15.1% were F4/80^+^ macrophages, and 12.4% were CD34^+^ CD64^+^ myeloid progenitor cells (Fig. 2, B to F). Notably, most (84%) of the MBV^+^ CD11b^+^ cells were CD64^+^/CD34^−^ (Fig. 2F). Consequently, we next performed gating to examine the proportion of NK cells, neutrophils, and macrophages that were CD64^+^. Almost all NK cells and macrophages (Fig. 2, G and I) and a high proportion of neutrophils (Fig. 2H) that were MBV^+^ also expressed CD64. This marker, also known as FcγRI, is an immunoglobulin G1 receptor that is typically expressed in later stages of granulocyte differentiation (*26*). While this marker offers an opportunity to identify cell subpopulations interacting with MBV, it remains unclear whether the expression of this marker is a consequence or a prerequisite for myeloid cells to uptake or interact with MBV. Although there was a tendency toward higher proportion of CD64^+^ cells after 24 hours of exposure to MBV compared to untreated animals, the only significant increase was observed in macrophages (fig. S3C), which does not confirm an increase of this marker as a consequence of MBV exposure. Distribution of MBV^+^ CD11b^+^ remained similar after 24 hours (Fig. 3, B to F), although the subpopulation of MBV^+^ CD11b^+^ Ly6G^+^ CD64^dim^ neutrophils increased after 24 hours of incubation with MBV (Fig. 3H). The fact that CD64^dim^ neutrophil populations have been related to neutrophil-committed progenitors (*29*) supports the hypothesis that MBV preferably interacted with myeloid progenitors. Moreover, a similar phenomenon was observed in NK cells after 24 hours (Fig. 3G) where a MBV^dim^ subset could be distinguished. However, these results do not clarify if the selective interactions of MBV with progenitors result in changes at phenotypic level in myeloid progenitors that differ from those observed in terminally differentiated myeloid subpopulations. We next investigated whether the phenotypic changes elicited by MBV were stable in myeloid progenitors and if these differed from those elicited on differentiated macrophages.

### MBV elicit epigenetic changes in myeloid cells that depend on the state of differentiation

**Fig. 4.**
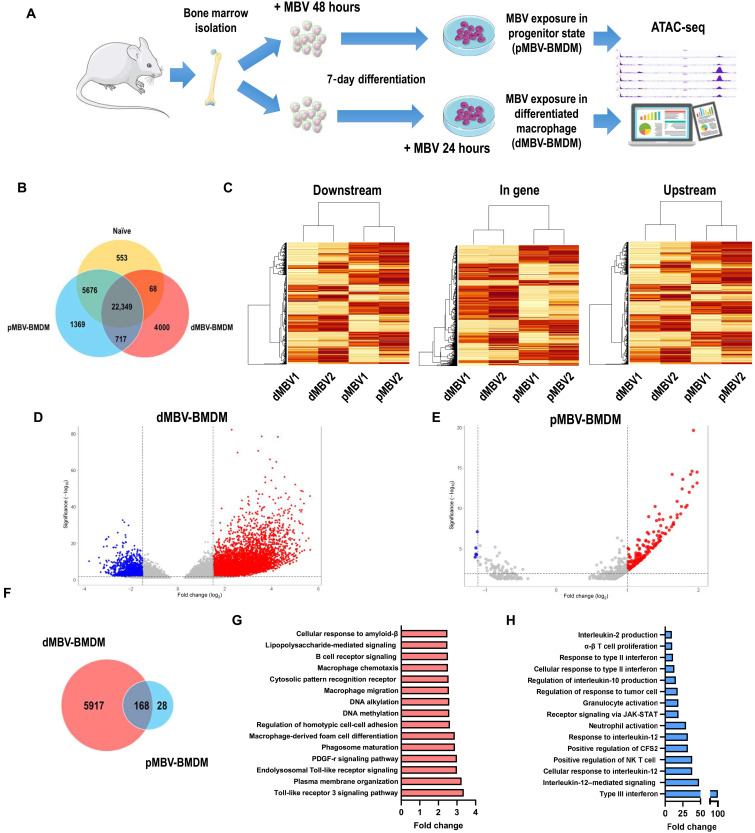
ATAC-seq analysis of BMDMs exposed to MBV in progenitor (pMBV-BMDM) state or after differentiation (dMBV-BMDM) show different effects at epigenetic level. Bone marrow monocyte cells were isolated from mouse femurs, differentiated, exposed to MBV either in progenitor state (pMBV-BMDM) or differentiated macrophage (dMBV-BMDM) state, and analyzed through ATAC-seq (**A**). Sequenced peaks present in both biological replicates (*N* = 2) of naïve, pMBV-BMDM, and dMBV-BMDM retrieved after ATAC-seq (**B**) were in their majority shared between the three groups, although both pMBV-BMDM and dMBV-BMDM showed a considerable number of exclusive peaks, indicating differences at epigenetic levels between samples. Significantly up-regulated and down-regulated peaks compared to naïve samples (DESeq log_2_ fold change, *P* < 0.05) in pMBV-BMDM or dMBV-BMDM were represented as heatmaps of upstream, downstream, and within gene regions (**C**), showing different epigenetic profiles between pMBV-BMDM and dMBV-BMDM. Volcano plots of epigenetically up-regulated and down-regulated genes (DESeq log_2_ fold change, *P* < 0.05) in both dMBV-BMDM (**D**) and pMBV-BMDM (**E**) showed a different profile, where dMBV-BMDM showed a larger amount of genes affected compared to pMBV-BMDM, although some genes were exclusive to pMBV-BMDM (**F**) and 168 were shared with dMBV-BMDM. The top 15 significantly GO term–enriched genes ordered by fold change in dMBV-BMDM (**G**) showed different profiles of genes compared to those observed in pMBV-BMDM (**H**). JAK-STAT, Janus kinase–signal transducers and activators of transcription. PDGF-r, Platelet-derived growth factor receptor; CFS2, Colony-Stimulating Factor 2.

The assay for transposase-accessible chromatin with sequencing (ATAC-seq) has recently been developed as a scalable tool to rapidly assess the epigenetically regulated chromatin accessibility at a whole-genome scale (*30*). Here, we carried out an ATAC-seq analysis on F4/80^+^ bone marrow–derived macrophages (BMDMs) after 7 days of differentiation in vitro, with exposure to MBV either at the progenitor stage (i.e., during the first 48 hours of differentiation, termed pMBV-BMDM) or at a terminally differentiated state (final 24 hours of differentiation, termed dMBV-BMDM) (Fig. 4A and fig. S4A). The goal was to determine whether MBV elicited different epigenetic changes in both stages of differentiation when compared to untreated naïve macrophages. The distribution of accessible chromatin between coding and noncoding regions was similar among the different conditions, although the dMBV-BMDM had a slightly higher percentage of peaks (*P* < 0.01) in noncoding regions (fig. S4B). The analysis showed 4000 peaks unique to the dMBV-BMDM group, 1369 peaks unique to the pMBV-BMDM group, and 553 peaks unique to the naïve group (Fig. 4B). Principal components analysis (PCA) on 14 variables—such as number of peaks, peaks in introns, peaks in exons, and peaks in CpG islands, among others (fig. S4E)—showed number of peaks as the largest contributor to the variability between treatments. PCA showed slight differences between naïve and pMBV-BMDM, while the separate clustering of dMBV-BMDM (fig. S4F) was explained almost entirely by the first principal component, which accounted for 99% of the variability. These results are indicative of different epigenetic profiles between naïve, pMBV-BMDM, and dMBV-BMDM, which was further explored by comparing regions in dMBV- and pMBV-BMDM that showed a significant increase or decrease in pairwise comparison with naïve macrophages by DESeq2 and shrunken log_2_ fold changes (*P* < 0.05).

Heatmaps of peaks with significant fold changes within coding regions (upstream, in-gene, and downstream) were plotted after row normalization (Fig. 4C) and revealed similar profiles between biological replicates within each group and further evidenced the differences between dMBV- and pMBV-BMDM. More variability between biological samples was present in downstream and upstream regions when compared to in-gene regions. Representation of these epigenetically modulated genes in volcano plots revealed that dMBV-BMDM presented a larger number of significantly up-regulated and down-regulated genes than pMBV-BMDM, and in both cases, the number of up-regulated genes was greater than those epigenetically down-regulated (Fig. 4, D and E). Further quantification and direct comparison confirmed that dMBV-BMDM presented a larger number of genes significantly up-regulated than pMBV-BMDM, which showed ~200 genes (Fig. 4F). This considerable difference in the number of epigenetic changes can be attributed to the lower amount progenitor myeloid cells interacting with MBV when compared to differentiated cells, as previously shown, and to the effect of differentiation on the epigenetic state of myeloid progenitors (*20*). Of the 196 genes in pMBV-BMDM for which accessibility was significantly up-regulated compared to naïve macrophages, 28 were unique to this group (i.e., did not overlap with dMBV-BMDM), confirming that MBV exposure can modulate the epigenetic profile of myeloid progenitors during the early stages of differentiation.

These epigenetically up-regulated genes in pMBV-BMDM included CD97, Zfp608, Dapk, eEf2,TNFS13b, and gngct, which are associated with hematopoiesis processes (*31*, *32*), regulation of tissue repair (*33*, *34*), and cross-talk between the adaptive and innate immune systems (*35*, *36*). A gene ontology (GO) enrichment for biological processes analysis (*37*) showed a significant enrichment of Toll-like receptor signaling, DNA modification (i.e., methyltransferases), macrophage phenotype, and cell adhesion GO terms in dMBV-BMDM (Fig. 4G), while in pMBV-BMDM, the most enriched GO terms were related to cytokines interferon-II (IFN-II), IFN-III, IL-2, IL-10, and IL-12 signaling, Janus kinase–signal transducers and activators of transcription pathway, and granulocyte differentiation regulation (Fig. 4H). These results demonstrate that the epigenetic effects of MBV treatment in myeloid cells vary depending on the differentiation state of the target cells. Further, GO enrichment of immunological processes (*38*) revealed that most of the epigenetically up-regulated genes related to leukocyte differentiation and adaptive and innate immune response regulation in dMBV-BMDM (fig. S5). One of the GO terms significantly enriched was the Fcrγ receptor signaling pathway, including the Fcrγ1 (CD64) and Fcrγ2b genes. This finding corroborates the flow cytometry results observed in Figs. 2 and 3 and supports the conclusion that MBV may be up-regulating these genes when interacting with macrophages. Of note, 168 genes were up-regulated in both dMBV-BMDM and pMBV-BMDM, which indicates that some of the MBV-elicited epigenetic changes in myeloid progenitors endure even after the epigenetic modifications associated with the differentiation process. Stated differently, a considerable degree of the epigenetic modulation of MBV on myeloid progenitors can be inherited by their descendant cells, while this exposure of progenitors to MBV induces few epigenetic changes exclusive to the progenitor state. GO term enrichment in immunological process analysis of up-regulated genes in both dMBV-BMDM and pMBV-BMDM (fig. S6) showed terms more related to leukocyte function regulation and monocyte-derived cells function, with several terms matching those observed in pMBV-BMDM (fig. S7). These observations suggest that the epigenetic changes elicited on pMBV-BMDM are maintained after differentiation and affect the function of these immune cells.

### Epigenetic changes elicited by MBV in myeloid progenitors and differentiated macrophages affect different sets of transcription factors

**Fig. 5.**
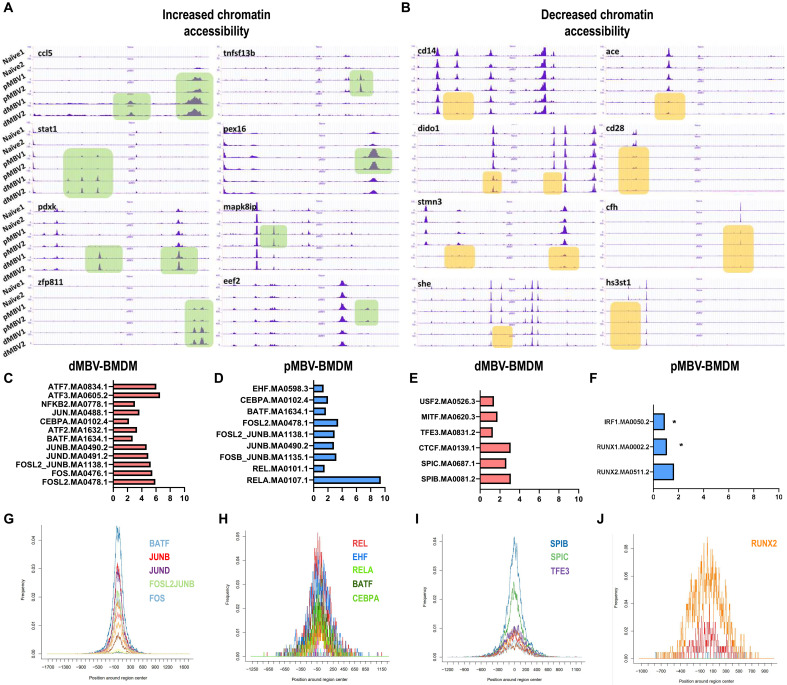
ATAC-seq peak and motif enrichment analysis in pMBV-BMDM and dMBV-BMDM proved differences in epigenetic modulation by MBV in myeloid progenitors and differentiated macrophages that affected immune-response–related transcription factor binding motifs. ATAC-seq peaks with significant differences between naïve and pMBV-BMDM and/or dMBV-BMDM (shrunken log_2_, DESeq2, *P* < 0.05) showed homogeneous responses between biological replicates and the presence of sequences with increased (green, **A**) and decreased accessibility (orange, **B**) in selected relevant genes for immune regulation, cell cycle, and metabolism. HOMER motif enrichment analysis of these sequences revealed the presence of motifs (hypergeometric *P* < 0.001) that matched (coefficient > 0.90) transcription factor binding motifs from JASPAR libraries (**C** to **F**). Regions with increased accessibility in the dMBV-BMDM group (C) and the pMBV-BMDM group (D) were enriched in binding sites for ATF, JUN, FOS, BATF, and CEBPA motifs, while in the pMBV-BMDM group, these sequences were also strongly enriched in RELA binding sites. Down-regulated accessibility sequences were enriched in CTCF, SPIC, and SPIB binding sites in dMBV-BMDM (E), while only RUNX2 matching motifs were found in pMBV-BMDM [(F), * indicates *P* > 0.05)]. Frequency of aligned motifs with respect to the center of the genomic regions analyzed was investigated to correlate high-frequency centered motifs with high likelihood motifs as binding sites for transcription factors (**G** to **J**). BATF, JUNB, JUND, and FOS presented the highest centered frequencies in increased accessibility sequences of dMBV-BMDM (G), while in pMBV-BMDM, these corresponded to REL, EHF, RELA, BATF, and CEBPA (H). Decreased accessibility sequences presented fewer significant matching motifs due to the lower number of overall sequences, although SPIC and SPIB were present in dMBV-BMDM (I) and RUNX2 in pMBV-BMDM (J).

The implications of the observed epigenetic changes in pMBV-BMDM and dMBV-BMDM were explored by analyzing the differences of chromatin accessibility between these groups at sequence level. Overall, chromatin accessibility was altered at locations both within genes and in upstream and downstream regions, mostly in noncoding intronic regions (Fig. 5, A and B), and with low variability between biological replicates. Relevant genes involved in inflammation and immune regulation such as the chemokine ligand *ccl5*, transcription factors such as *mapk8ip* and *stat1*, or cell cycle regulatory factors such as *tnfsf13b* or *eef2* presented higher ATAC-seq peaks in dMBV-BMDM and/or pMBV-BMDM compared to naïve BMDMs (Fig. 5A). Few regions of decreased chromatin accessibility were observed in the pMBV-BMDM group, in contrast to the dMBV-BMDM group (Fig. 5B). While the reasons for this are unclear, it is possible that regions that are repressed by MBV treatment at the progenitor stage are then reexposed during the differentiation process. A few exceptions such as *cd28* and *chf*, intimately involved in the inflammatory reaction, showed decreases in both pMBV-BMDM and dMBV-BMDM. Other genes related to disease progression and immunomodulation such as *ace*, *cd14*, and *she* or to metabolism and cell cycle such as *dido1* and *stmn3* showed decreased chromatin accessibility in dMBV-BMDM. These changes in peak distribution put in evidence the regulatory function of MBV in myeloid cells but with different mechanisms in function of their stemness.

To further understand the mechanisms and pathways that are involved in these epigenetic changes, a motif analysis was conducted using Hypergeometric Optimization of Motif EnRichment (HOMER) (*39*) in those sequences with intervals significantly (DESeq2, *P* < 0.05) up-regulated and down-regulated in pMBV-BMDM and dMBV-BMDM compared to naïve BMDMs. A list of the top-ranked up-regulated and down-regulated motifs in each sample is shown in table S1. These top-ranked motifs were further explored and compared to known motifs available in public libraries (i.e., JASPAR), and those known motifs with a correspondence coefficient above 0.90 were selected to visualize the motif enrichment within the up-regulated and down-regulated genomic regions using the freeware TFmotifView (*40*). Those motifs with a significant representation (hypergeometric *P* < 0.001, unless indicated) among these regions of interest were plotted in function of their fold change with respect to control regions globally (Fig. 5, C to F). Regions with increased chromatin accessibility were enriched for binding motifs for FOS, JUN, ATF, and BATF, both in dMBV-BMDM and pMBV-BMDM, and strong enrichment for RELA binding motifs was observed in the pMBV-BMDM group only (Fig. 4, C and D). A lower number of motifs within down-regulated accessibility sequences were observed. dMBV-BMDM down-regulated accessibility sequences showed motifs matching with CTCF, SPIC, and SPIB (Fig. 5E), while pMBV-BMDM showed RUNX2 as the only statistically significant (*P* < 0.001) matching motif (Fig. 5F).

We further aligned these motif sequences to the genomic regions that correspond to binding sites for transcription factors in dMBV-BMDM and pMBV-BMDM generated genomes. In these alignment analyses, a centered high frequency indicates a high probability for these sequences corresponding to transcription factor binding sites (*40*). BATF, JUND, JUNB, and FOS motifs were those with the highest centered frequencies in dMBV-BMDM (Fig. 5G), indicating that these transcription factors are the most likely to be binding the newly accessible chromatin regions. In the case of pMBV-BMDM (Fig. 5H), the transcription factors REL, EHF, RELA, and BATF presented the highest frequencies aligned to the center of the genomic regions. The highest-frequency motifs in regions with down-regulated accessibility in dMBV-BMDM corresponded to SPIC and SPIB (Fig. 5I), while only RUNX binding sites were enriched in pMBV-BMDM (Fig. 5J). The fact that MBV could be regulating cell function by changing accessibility of genomic regions to different sets of transcription factors in dMBV-BMDM and pMBV-BMDM could indicate that MBV affect myeloid cells differently in function of their state of differentiation.

### MBV epigenetic and phenotypic effects in vivo depend on the delivery route

**Fig. 6.**
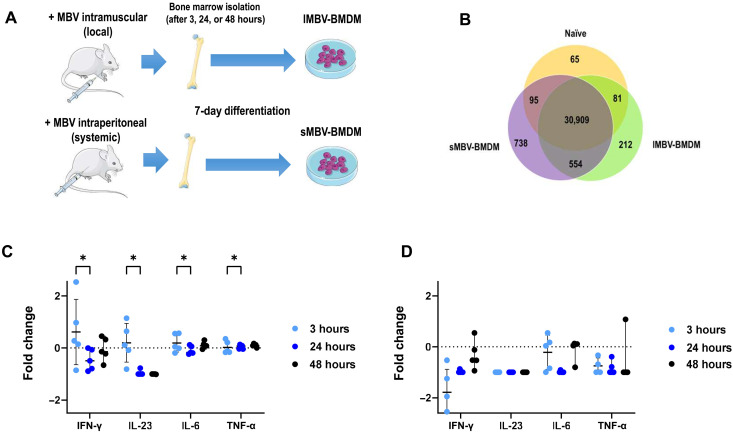
Epigenetic and phenotypic effects in vivo of one MBV dose in BMDMs. Mice were injected intraperitoneally (systemic) or intramuscularly (local) with MBV, bone marrow was collected after 3, 24, or 48 hours, and cells were differentiated and used for analysis (**A**). ATAC-seq was performed on cells from bone marrow collected after 24 hours with a single 10^11^ MBV dose. Analysis of distinctive peaks (**B**) in each condition showed unique peaks in sMBV-BMDM, lMBV-BMDM, and naïve BMDM, in decreasing order (results showed as averages of *N* = 2). Bone marrow from mice injected locally (**C**) and systemically (**D**) with 10^11^ MBV (100-μl dose systemically, 50 μl locally) was collected 3, 24, or 48 hours after injection, differentiated for 7 days, and challenged with LPS in vitro, and inflammatory cytokine production was measured. In local delivery (C), IFN-γ showed a decrease in the 24 hours groups, while IL-23 did so in 24 and 48 hours groups with the highest doses. IL-6 and TNF-α showed minimal effects. For BMDMs from mice treated with MBV systemically (D), IL-23 production was blocked in all groups and doses. IL-6 production was lower in the 24 hours group, while slight down-regulation effects in TNF-α were observed at all time points (*N* = 5, data as average and SD, *P < 0.05).

The implications of the epigenetic effect of MBV on myeloid cells were evaluated in vivo. A single injection of MBV was administered IP or IM (local) in mice. Bone marrow cells were then isolated and differentiated to macrophages, and ATAC-seq analysis was performed (Fig. 6A). PCA (fig. S8) showed differences between the IP-delivered MBV (sMBV-BMDM) group and sham treatment BMDM samples, but the locally injected MBV group (lMBV-BMDM) did not show differences with any of the other groups (fig. S8). The number of unique peaks in sMBV-BMDM and lMBV-BMDM (Fig. 5B) was lower than those observed after exposure of myeloid cells to MBV in vitro. DESeq2 analysis did not retrieve any significant differences between sham and sMBV-BMDM nor lMBV-BMDM. This relatively low effect could be explained by several factors such as the fast clearance of MBV in vivo, the relatively low uptake of MBV by myeloid progenitors, and the dose.

The response to inflammation of macrophages exposed to a single dose of 10^11^ MBV in vivo was assessed after differentiation in vitro and challenge with lipopolysaccharide (LPS). In each condition and time point, the fold change in M1-type cytokine production compared to the phosphate-buffered saline (PBS)–injected control group was calculated. Local delivery MBV elicited a decrease in IFN-γ and IL-23 production (Fig. 6C). IL-6 and TNF-α showed no significant decrease nor increase in the ratio of cytokine production by challenged macrophages. IP delivery had a more noticeable effect on the phenotype of macrophages, which could be explained by a greater amount of MBV reaching the bone marrow, as previously noted. IFN-γ (Fig. 6D) showed a marked decrease in BMDMs collected 3 hours after MBV injection, while this effect decreased with time. IL-23 production was prevented by MBV systemic delivery at all time points (Fig. 6H). IL-6 showed a decrease in production that peaked after 24 hours and then faded, while the TNF-α ratio decreased within 24 hours. Overall, a single dose of MBV appeared to prevent down-regulation of the expression of IL-23 and IFN-γ, whereas this effect was more noticeable in systemic delivery. The down-regulation of IL-23 and IFN-γ observed at the protein level did not match the results of reverse transcription quantitative polymerase chain reaction (RT-qPCR) at the same time point (fig. S8, A and B), which could suggest a posttranslational regulation rather than a modulation at the transcript level. On the other hand, the trends observed in TNF-α and IL-6 RNA expression were similar to those observed at the protein level (fig. S8, C and D). Considering that all these effects of in vivo MBV administration on BMDMs were observed more than 7 days after harvest and in vitro differentiation, these results show that there is an effect of a single MBV dose on myeloid progenitors, but this varies depending on time of exposure and routes of delivery. The discrepancies with ATAC-seq analysis suggest that other factors such as dose concentration can also influence the epigenetic effect of MBV in vivo.

**Fig. 7.**
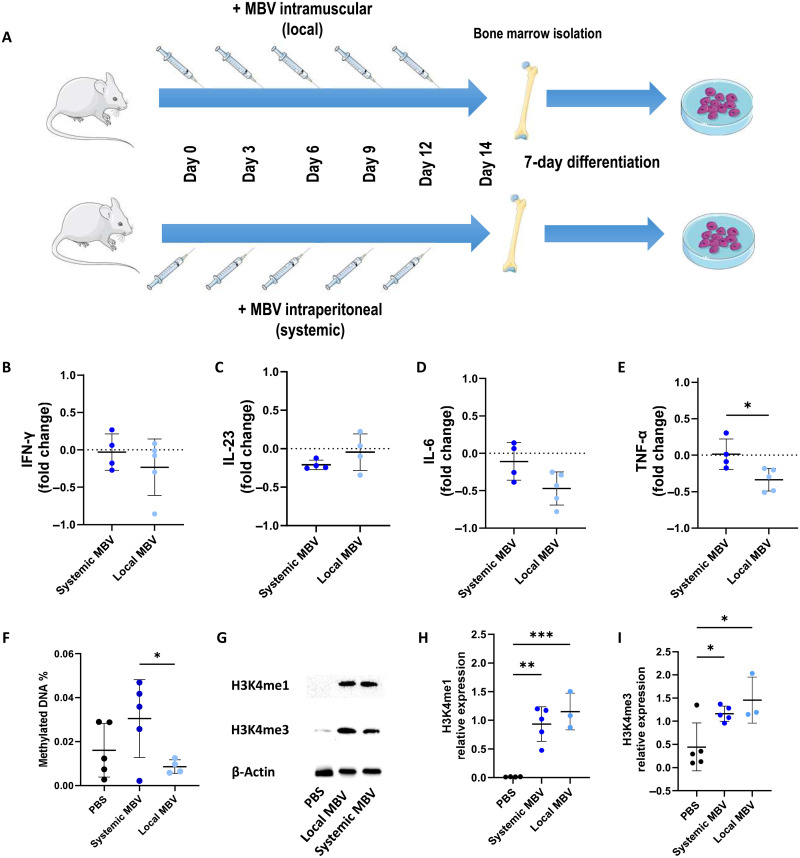
Epigenetic and phenotypic effects in vivo of five doses of 10^8^ MBV doses in BMDMs. Bone marrow from mice treated with five doses of 10^8^ MBV during 2 weeks was collected 48 hours after the last injection, differentiated into macrophages (BMDMs), and challenged in vitro with LPS (**A**). Assessment of cytokine production showed minimal effect in IFN-γ release (**B**), while a slight decrease in IL-23 (**C**) was observed in the systemic MBV group. Both IL-6 (**D**) and TNF-α (**E**) production was lower in the local MBV groups. Total DNA methylation (**F**) was slightly higher in BMDMs from the systemic MBV-treated group. Western blot of H3K4me1 and H3K4me3 showed an increase of histone methylation in both MBV groups (**G**), which was confirmed by densitometry analysis in both H3K4me1 (**H**) and H3K4me3 (**I**) (*N* = 5, data as average and SD, *, **, and *** indicate *P* < 0.05, *P* < 0.01, and *P* < 0.001, respectively).

While single dose of MBV had noticeable effects in the phenotype of macrophages, ATAC-seq results showed that this effect is limited at the tested dose. Given that previous studies showed a prominent immunomodulation by MBV at several dose regimens (*16*, *17*), the phenotypic and epigenetic effects were determined by repeated dosing of MBV at lower concentrations (10^8^ MBV). Cytokine production was evaluated by challenged BMDMs isolated from mice undergoing injection of five doses of 10^8^ MBV every 3 days (Fig. 7, B to E). While IFN-γ (Fig. 7B), IL-6 (Fig. 7D), and TNF-α (Fig. 7E) showed a decrease in the ratio of cytokine production in the local MBV delivery group, the IL-23 ratio (Fig. 7C) showed a slight down-regulation in systemic delivery. Nonetheless, the only significant differences observed were between local and systemic delivery in TNF-α production (*P* < 0.05). None of these trends were observed in the RT-qPCR results (fig. S9, E to H). These findings suggest that the modulation of epigenetic and phenotypic hallmarks in myeloid progenitors after MBV repeated dosing is dependent on the route of administration. Also, the observations with MBV single dosing may indicate differences with repeated dosing given the cumulative effect. Since ATAC-seq showed weak trends after a single highly concentrated dose of MBV, the epigenetic effects of MBV after repeated doses was investigated by measuring the total methylation in DNA and levels of monomethylation (H3K4me1) and trimethylation (H3K4me3) of the histone H3K4, well-known epigenetic modulation features in monocytes (*20*). Total DNA methylation showed higher values after repeated local delivery of MBV (Fig. 7F), although the only significant differences were found between local and systemic delivery (*P* < 0.05), and no differences with PBS control were observed. However, histone methylation (Fig. 7G) showed an increase after both systemic and local delivery, which was confirmed after quantification for both H3K4me1 (Fig. 7H, *P* < 0.01 and *P* < 0.001 with PBS in local and systemic delivery respectively) and H3K4me3 (Fig. 7I, *P* < 0.05 with PBS in both delivery routes), confirming the enduring epigenetic effect of MBV on monocytes after isolation and differentiation in vitro.

## DISCUSSION

The study of the epigenetic effects of EV on immune cells has increased in recent years, particularly in studies of mesenchymal stem cell–derived small EV (*21*, *23*, *24*). The present study showed that MBV derived from urinary bladder matrix (UBM) ECM induce epigenetic changes in myeloid cells and that the capacity for MBV-based modifications differ between progenitor and terminally differentiated myeloid cells (i.e., macrophages). These findings advance our understanding of the mechanisms by which ECM-based biomaterials influence tissue healing events when used as bioscaffolds. These findings also suggest therapeutic immunomodulatory approaches for a variety of diseases in which the immune system plays a central role.

The present study showed that murine bone marrow myeloid cells, including progenitors, uptake MBV in vitro, and that following in vivo administration, a high signal of lipid-tagged (near infrared, DiD) MBV is present in the bone marrow. Notably, IP injection resulted in the highest signal at 24 hours, likely due to a balance between rapid distribution (as seen in IV) and local retention (maximized in IM delivery) (*25*). In vitro data confirmed that CD34^+^/CD64^+^ myeloid progenitors uptake MBV, supporting the hypothesis that MBV exposure could influence the phenotype of their differentiated progeny, a process referred to as innate memory (*20*, *41*). However, despite attempts to track CFDA-SE–labeled MBV in vivo, no signal was observed in bone marrow flushes (fig. S10). While DiD is well suited for in vivo imaging and avoids tissue-generated background, the instrumentation used for flow cytometry in the present study did not include near-infrared detection capability. In addition, uptake of CFDA-SE–tagged MBV and transport into the cell cytoplasm result in cleavage of CFSE by intracellular esterases to generate signal (*42*). However, this cleavage may also result in an underrepresentation of MBV uptake both in vitro and in vivo. On the other hand, cytosolic cleavage could represent another layer of specificity for MBV uptake, indicating rather the need of esterase cleavage to demonstrate internalization or a possible specific sublocalization of MBV components upon uptake. Nonetheless, little is known about the intracellular uptake of MBV to date, and EV uptake machinery varies as a function of EV composition (*5*). While UBM-derived MBV accumulation in secretory organs such as kidney and liver has been previously reported (*25*), in vivo results of the present study show the successful delivery of MBV to the bone marrow after injection, confirming the accumulation in bone marrow also reported in a more recent in vivo study (*14*). In addition, flow cytometry confirms interaction of MBV with myeloid progenitors after in vitro treatment. However, future studies are needed to confirm the accumulation of MBV in the bone marrow and their relevance to the observed immune effects. It is possible that these immunomodulatory effects are due to systemic signaling in response to MBV injection. To this end, bone marrow transplants from mice treated with MBV into naïve mice could be used as a model to test this hypothesis.

ATAC-seq analysis confirmed the interaction of MBV with myeloid cells in vitro, with a more pronounced effect on differentiated macrophages (dMBV-BMDM) compared to progenitor cells (pMBV-BMDM). While the number of epigenetically modulated regions was lower in pMBV-BMDM, the presence of such regions suggests that MBV treatment of progenitors may influence the phenotype of their differentiated progeny. This hypothesis is supported by previous studies reporting phenotypic modulation of macrophages more than 50 days after in vivo MBV administration (*14*, *16*); a finding in contradiction to the fact that terminally differentiated macrophages typically have a short life span (i.e., 2 to 3 days) (*11*). Modulation of cell cycle and apoptotic processes has been described in myeloid cells under certain conditions (*43*), and some of the genes found to be epigenetically modulated in the present study (e.g., *eef2*) are closely linked to differentiation and the cell cycle (*33*). Further studies to explore the effects of MBV on myeloid cells at the epigenetic, transcriptomic, and protein levels are needed to understand the mechanisms of sustained phenotypic immunomodulation. The ATAC-seq results together with the cytokine response analysis suggest a dual effect of MBV: an effect down-regulating proinflammatory markers and promoting proremodeling markers (*6*, *8*), followed by an innate memory effect, as indicated both in this study and in previous work (*15*, *16*).

Epigenetic analysis revealed chromatin accessibility changes in IFN and IL-12, while other common inflammatory markers such as TNF-α and IL-6 did not show a direct epigenetic modulation. These results contrast with the ex vivo cytokine challenge experiments, highlighting the complexity of the regulatory pathways that govern innate memory (*41*). Transcription factor analysis showed that MBV exposure increased chromatin accessibility to BATF, JUN, and FOS while down-regulating accessibility for SPIC and SPIB. These genes are involved in macrophage polarization, response to inflammation, and graft rejection (*44*–*47*), processes previously implicated in ECM bioscaffold–mediated immunomodulation (*44*, *48*, *49*). BATF also plays a role in regulation of T cells, B cells, and myeloid antigen-presenting cell interactions (*45*, *50*), consistent with the GO term analysis. BATF binding motifs were also made accessible in pMBV-BMDM, suggesting that MBV induce stable epigenetic modifications in progenitor cells that persist after differentiation. These results align with recent work by Bae *et al.* (*22*), who showed that BATF accessibility is linked to RANKL-mediated bone resorption by osteoclasts, which is potentially relevant to the observed regulatory function of MBV in a rheumatoid arthritis model (*16*) or in bone regeneration as recently reported (*14*). REL and RELA transcription factor binding motifs were also made more accessible in pMBV-BMDM, implicating the nuclear factor κB signaling axis in modulation of inflammation and cell cycle processes (*51*).

Despite observation of robust epigenetic effects after MBV treatment in vitro, in vivo findings showed limited effects, with no significant differences between MBV-treated and naïve macrophages after ATAC-seq analysis. This finding may be due to the lower amount of MBV reaching the bone marrow myeloid cells in vivo compared to in vitro exposure. Future studies could address this limitation using enrichment techniques such as flow sorting or cell separation columns. However, certain differences in macrophage response to inflammatory challenges could be observed after one MBV dose in vivo, indicating a potential epigenetic effect. In addition, the impact of single versus multiple MBV dose regimen was evaluated in vivo. In local delivery with a single dose, most cytokines showed no modulation in the 48-hour postinjection groups, in contrast to the groups receiving multiple local injections. This effect could account for a sustained release of MBV and accumulation of epigenetic changes or by an indirect sustained effect by tissue-resident macrophages, which have a longer life span (*11*, *43*). The epigenetic effects of repeated dosing with MBV were confirmed by an increase in the monomethylation and trimethylation of histone H3K4, a key epigenetic regulatory mark in macrophages (*20*) which could represent a future target for chromatin immunoprecipitation sequencing analysis to further understand the mechanisms behind the epigenetic modulation of MBV.

Overall, these findings show that the epigenetic effects of MBV depend on dose regimen and route of administration. Future studies are required to optimize these conditions for specific applications in regenerative medicine where MBV have already shown a sustained immunomodulatory effect. In addition, future studies using human bone marrow ex vivo would add insight as to the relevance of the current findings toward the clinical translation of MBV as an immunomodulatory therapeutic tool.

The present study provides previously unknown insights into the effects of MBV treatment on myeloid cell epigenetics, as opposed to previous studies that only considered a direct effect by integrin-mediated signal transduction or, more recently, noncanonical signaling of ECM-resident cytokines. These observations suggest the possibility for the use of ECM immunomodulatory effects as a long-lasting therapy. Nonetheless, several questions remain, including the mechanisms of MBV uptake by cells (*25*) and the molecular machinery involved. The specific components of the MBV responsible for epigenetic modulation remain to be identified as well. GO term analysis showed involvement of methyltransferases in mediating the observed effects, but other potential cargo within MBV could also play a role in macrophage modulation (*52*, *53*). Comprehensive characterization of MBV protein, nucleic acid, and lipid cargo will be required to ascertain these mechanisms. Last, the extent to which ECM bioscaffold implantation results in MBV release and downstream epigenetic effects remains unknown. While the dose of MBV that an ECM scaffold contains is approximately equivalent to the doses of MBV concentrations tested in the present study, other factors such as gradual MBV release (i.e., slow during ECM degradation versus burst release of MBV injection) must be considered. The present study was also limited to monocyte progenitors and macrophages, leaving for future studies the exploration of MBV epigenetic effects on other myeloid progenitors (i.e., neutrophil precursors) and terminally differentiated cells. Last, the mechanisms by which MBV induce epigenetic changes and their duration are not identified, in the present work. Future studies such as an adoptive transfer of bone marrow between MBV-treated mice and irradiated recipients or pulse-chase studies may help address such mechanisms.

In summary, the present study demonstrates that MBV reach the bone marrow after local and systemic injection in mice, where they are taken up by myeloid cells, including CD64^+^/CD34^+^ myeloid progenitors. In addition, MBV elicited epigenetic modifications in myeloid cells that depend on the differentiation state (i.e., progenitor state or terminally differentiated macrophages) and the number of doses and route of administration. Preliminary results also suggest that these epigenetic modifications involve the methylation of the histone H3K4. These epigenetic regulations elicited by MBV suggest their potential long-lasting therapeutic use and a step forward in our understanding of ECM functions.

## MATERIALS AND METHODS

### UBM MBV isolation

Porcine UBM tissue was decellularized by scraping, delamination, and treatment with ethanol/peracetic acid (*7*). Then, UBM was lyophilized, micronized, and hydrolyzed with Liberase (Sigma-Aldrich, USA) in tris-HCl buffer overnight at 37°C with continuous agitation. MBV were then isolated by serial centrifugation steps for 10 min at 500*g*, 20 min at 2500*g*, and 30 min at 10,000*g*, followed by ultracentrifugation at 100,000*g* for 70 min. MBV concentration was assessed by nanoparticle tracking analysis with a NanoSight NS500 (Malvern Panalytical, USA) where *N* = 3 captures at a dilution that yields readings of 20 to 200 vesicles per frame, for a total of 30 s per capture was performed. MBV were further characterized using an exosome marker array (System Biosciences, USA).

### MBV staining, injection, and bone marrow flush imaging

All animal studies were carried out under the supervision and approval of the University of Pittsburgh Institutional Animal Care and Use Committee (protocol #22010381). MBV were tagged with a lipidic near-infrared fluorescent tag (ExoGlow, System Biosciences, USA) following the manufacturer’s instructions. Then, MBV solutions in sterile PBS with 1 × 10^11^, 1 × 10^8^, or 1 × 10^6^ total particles (in 100 μl volume for systemic delivery or 50 μl for local injection) were injected IP, IV, or IM in Balb/c mice (*N* = 5; the Jackson Laboratory, USA), using PBS-treated with fluorescent tag as control. MBV concentrations were chosen on the basis of a previous study (*16*). Then, animals were euthanized after 3 or 24 hours, femurs were dissected, and bone marrow was collected by centrifugation as previously described (*54*). Bone marrow pellets were then resuspended by repeated pipetting in 1 ml of sterile PBS and transferred to a 24 well-plate for fluorescent imaging in an in vivo imaging system IVIS (PerkinElmer, USA). Then, signal was quantified using the IVIS software by measuring the average radiance efficiency per well. Since the greatest accumulation was observed with 10^11^ MBV dose, this dose was chosen for the rest of experiments.

For whole blood analysis, 200 μl was collected after euthanasia and heart crush and then treated with red blood cell lysis buffer for 5 min at 4°C, and leukocytes were collected after centrifugation at 500*g* for 5 min at 4°C. The cell pellet was then resuspended in 1 ml of sterile PBS and imaged in the IVIS system. A positive control of 10^12^ tagged MBV per milliliter was imaged after 48 hours of incubation in the dark at 4°C, to ensure the stability of the signal.

### Flow cytometry analysis

Bone marrow from Balb/c mice were collected and resuspended in PBS as described above (*N* = 3) and placed in six-well plates. MBV were tagged with CFSE (CFDA-SE) and used to treat bone marrow flushes at 1 × 10^11^ particles/ml for 3 or 24 hours in vitro at 37°C and 5% CO_2_ in the dark. Bone marrow flushes were then centrifuged at 500*g*, washed in PBS, and resuspended in flow cytometry buffer (0.2-μm filtered 3% bovine serum albumin in PBS). Cells were then stained with an antibody panel developed in-house (Table 1).

**Table 1.** Panel of antibodies for MBV uptake identification in bone marrow isolates. FITC, fluorescein isothiocyanate; PE, phycoerythrin; APC, Allophycocyanin; BV, Brilliant violet; AF, Alexa FluorPer; CP, Peridinin-Chlorophyll-Protein.

Antibody	Target	Dye	Manufacturer and catalog no.	Dilution
CSFE	MBV	FITC	Thermo Fisher Scientific, C1157	1 x 10^11^ p/ml
CD11b	Myeloid cells	PE	eBioscience, 12-0112-82	1:800
CD34	Myeloid progenitors	AF700	Invitrogen, 56-0341-82	1:25
CD64	Myeloid progenitors	APC	Invitrogen, 17-0641-82	1:25
F4/80	Macrophages	PerCP-Cy5.5	eBioscience, 17-4801-82	1:50
CD4	T cells	BV-395	Invitrogen, 363-0042-82	1:800
CD335	NK	PE Cy7	Invitrogen, 25-3351-82	1:25
Ly6G	Neutrophils	APC-eF780	Invitrogen, 47-5931-82	1:800

After staining, cells were fixed with 2% paraformaldehyde (PFA) in flow cytometry buffer for 5 min at room temperature, and cells were analyzed through flow cytometry. Flow cytometry was carried out with a BDFACS Aria II using the software BD FACSDiva 9.0.1 (BD Biosciences, USA), where fluorescence-minus-one controls were used for compensation of each antibody/dye. Viability dye (eBioscience, USA) was used to confirm a >90% survival of cells. Control bone marrow flushes (*N* = 3) were stained with the same protocol without incubation with MBV. Analysis of results was performed with FlowJo software (FlowJo LLC, USA).

For in vivo uptake analysis via flow cytometry, CFDA-SE–labeled MBV were injected IV or IP at 1 × 10^13^ particles/ml for a total of 1 × 10^12^ MBV per mouse (*N* = 3). At 3 and 24 hours, bone marrows were collected, centrifuged, fixed, and stained as indicated above.

### ATAC-seq analysis

#### Cell isolation and MBV exposure

An ATAC-seq analysis was conducted to assess the changes in chromatin accessibility in differentiated BMDMs exposed to MBV either in myeloid progenitor state or differentiation stage. BMDMs were isolated as described above and incubated at 0.5 × 10^6^ cells/cm^2^ in BMDM differentiation medium, which consisted of high-glucose Dulbecco’s modified Eagle’s medium (DMEM; Gibco, USA), 10% fetal bovine serum (FBS), 10% L929 fibroblast supernatant, 0.1% β-mercaptoethanol (Sigma-Aldrich, USA), 1% penicillin/streptomycin (PS), 10 mM nonessential amino acids (Gibco, USA), and 10 mM Hepes buffer. After 24 hours, the progenitor state exposure to the MBV group (pMBV-BMDM) was treated with MBV at 10^11^ MBV/ml for 24 hours, washed in PBS, and incubated in BMDM differentiation medium to complete macrophage differentiation for 6 more days and in growth medium (DMEM, 10% FBS and 1% PS) for 24 hours more. For the differentiated state exposure to MBV (dMBV-BMDM), cells were differentiated into BMDMs for 7 days. Then, they were incubated with MBV at 10^11^ MBV/ml for 24 hours in growth medium. For in vivo–treated groups, mice were injected with 10^11^ MBV whether IP (systemic, sMBV-BMDM) or IM (local, lMBV-BMDM), and after 24 hours, bone marrow was isolated, and differentiation of BMDMs was carried out equally to previous groups without further treatment with MBV. Then, all groups were treated with trypsin for 5 min at 37°C after a PBS wash and gently detached by repeated pipetting. Later, cells were treated with growth medium, centrifuged at 500*g* for 5 min, resuspended at 200,000 cells/ml in 50% FBS/40% growth medium/10% dimethyl sulfoxide, and stored at −80°C. A total of 100,000 cells per replicate were used for ATAC-seq analysis. Cells without MBV treatment that underwent the same differentiation process were used as control.

#### Immunolabeling

To verify BMDM phenotype, a subset of cells from each group was fixed in 4% PFA for 5 min at room temperature and stained through immunocytochemistry. Briefly, fixed cells were blocked with 5% normal goat serum and 0.1% Triton X-100 in PBS for 1 hour at room temperature, incubated with 1:100 anti-F4/80 antibody (Thermo Fisher Scientific, USA) 1 hour at room temperature, washed three times with PBS, and incubated with Alexa Fluor 488 anti-rabbit secondary antibody for 1 hour at room temperature. After further washing with PBS, nuclei were stained with DAPI for 5 min, and cells were imaged using a live-cell microscope (Observer.Z1, Zeiss, USA). Image analysis was carried out using CellProfiler (Broad Institute, USA) to quantify the percentage of positive F4/80 cells.

#### ATAC-seq data analysis

ATAC-seq analysis was performed by ActiveMotif (California, USA), using proprietary protocols and Encyclopedia Of DNA Elements (ENCODE) guidelines (*55*). For bioinformatic analysis, RStudio (Posit Software, USA) was used to perform PCA and generate heatmaps. For pairwise comparisons, DESeq2 and shrunken log_2_ fold changes were used as statistical analysis where *P* < 0.05 was considered as statistical significance and which results were used for volcano plot generation and further analysis. The UCSC browser online tool was used for peak visualization, whereas GO enrichment analysis was performed with the online resources GO enrichment tool (Global Core Biodata Resource, USA) (*37*) and GONet for immunological process enrichment diagram analysis (*38*). Motif analysis in significant different regions was carried out by HOMER analysis (*39*), whereas distribution and alignment of motifs were analyzed using the freeware TFmotifView (*40*).

### MBV-primed BMDM challenge ex vivo

#### MBV dosing, cell isolation, and challenge ex vivo

We further investigated the effects of dosing and number of doses in the phenotype of BMDMs after local (IM) and systemic (IP) administration. For each route, we compared the effect of a single dose of 10^11^, 10^8^, or 10^6^ total MBV (100 μl in systemic delivery, 50 μl in local injection) on BMDMs collected after 3, 24, and 48 hours from the injection and differentiated for 7 days as described above. Only effects with 10^11^ MBV dose are shown. For repeating dosing comparison, five doses (one every 3 days) of 10^8^ MBV were administered IM or IP, bone marrow was collected 48 hours after the last injection, and BMDMs were differentiated as previously described. Once differentiated, BMDMs were challenged in vitro with LPS of *Escherichia coli* (100 ng/ml; Sigma-Aldrich, USA) for 24 hours, media was collected for enzyme-linked immunosorbent assay analysis, and cell layers were collected using TRIzol (Thermo Fisher Scientific, USA) reagent for RNA isolation.

#### Cytokine and gene expression analysis

Cell medium was tested to quantify the presence of IFN-γ, TNF-α, IL-6 (BioLegend, USA), and IL-23 (R&D Systems, USA) following the manufacturers’ protocols. The fold change of challenged macrophage cytokine production increment with respect to that observed after PBS injection in the same conditions was calculated as follows

$$\text{Fold change in cytokine production}=\frac{\left(\text{MB}V_{M1}-\text{MB}V_{M0}\right)-\left(\text{PB}S_{M1}-\text{PB}S_{M0}\right)}{\left(\text{PB}S_{M1}-\text{PB}S_{M0}\right)}$$

where M1 and M0 values correspond to cytokine readings with and without LPS challenge, respectively.

After RNA isolation with the TRIzol protocol and reverse transcription (High-Capacity cDNA reverse transcription, Thermo Fisher Scientific, USA), gene expression was measured by RT-qPCR using TaqMan probes for TNF-α (Mm00443258_m1), IL-6 (Mm00446190_m1), IL-23 (Mm00518984_m1), and IFN-γ (Mm01168134_m1), with hypoxanthine-guanine phosphoribosyltransferase (Mm03024075_m1) as the housekeeping probe (all probes were purchased from Thermo Fisher Scientific, USA).

#### DNA and histone methylation analysis

Following the TRIzol protocol, DNA and proteins were further isolated. DNA was quantified by NanoDrop (ND-1000, Thermo Fisher Scientific, USA), and methylated DNA percentage was calculated with a DNA methylation quantification kit (Epigentek, USA). Protein was quantified using a Bicinchoninic Acid (BCA) Assay kit (Thermo Fisher Scientific, USA), and Western blot was performed to measure histone methylation. Briefly, 5 μg of total protein was loaded in each well of a precasted SDS–polyacrylamide gel electrophoresis (SDS-PAGE) 4 to 20% gradient gel plate (Bio-Rad, USA), and SDS-PAGE was performed at 120 V. Later, proteins were transferred to a polyvinylidene difluoride membrane, and membranes were blocked for 1 hour with 5% skimmed milk in tris-buffered saline 0.1% Tween 20 (TBST; Santa Cruz Biotechnology, USA) and incubated overnight at 4°C with 1:1000 dilutions of primary anti-H3K4 monomethylated histone and anti-H3K4 trimethylated histone antibodies (Epigentek, USA). Later, blots were washed three times in TBST and incubated in anti-rabbit horseradish peroxidase secondary antibody (Dako, USA) at 1:2000 dilution for 1 hour at room temperature. After three further washes in TBST, signal was developed using an enhanced chemiluminescence substrate (Bio-Rad, USA), and chemiluminescence was measured with a Chemi-Doc measuring system (Bio-Rad, USA). After antibody stripping, the procedure was repeated with β-actin (Cell Signaling Technology, USA) primary antibody, which signal was used to normalize methylated histones readings.

### Statistical analysis

Unless otherwise indicated, statistical analysis was performed with GraphPad Prism 9 (GraphPad Holdings LLC, California, USA). Normal distribution of data was assessed using a Shapiro-Wilk test. One-way analysis of variance (ANOVA) and Fisher’s least significant difference post hoc analysis were used to assess statistical significance for normally distributed data, which were assumed when *P* < 0.05. Kruskal-Wallis and Dunn’s post hoc nonparametric tests were used for nonnormally distributed data. For paired test, normal data were compared using a two-tailed *t* test, whereas a Wilcoxon test was used for nonnormal data. All data are expressed as average ± SD.
