# Supplementary material for: Matrix-bound nanovesicles as epigenetic modulators of myeloid cells
Source: Sci Adv. 2026 Feb 11;12(7):eadx9159. doi: 10.1126/sciadv.adx9159 (PMC12893231; doi:10.1126/sciadv.adx9159)
Supplement: Supplementary file 1 — Figs. S1 to S10 Table S1 Data S1 [file sciadv.adx9159_sm.pdf]

Science Advances
AAAS

Supplementary Materials for

Matrix-bound nanovesicles as epigenetic modulators of myeloid cells

Héctor Capella-Monsonís *et al.*

Corresponding author: Stephen F. Badylak, sfb8@pitt.edu

Sci. Adv. **12**, eadx9159 (2026)
DOI: 10.1126/sciadv.adx9159

This PDF file includes:

Figs. S1 to S10
Table S1
Data S1

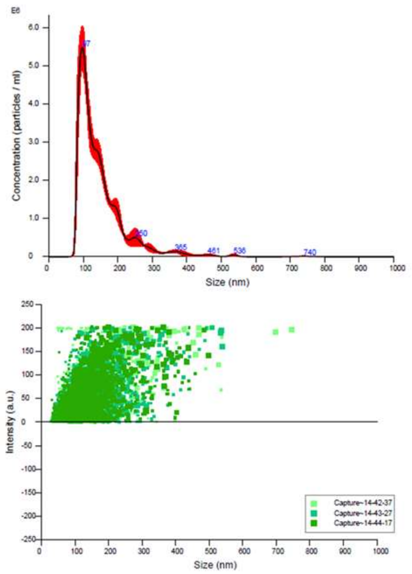

Results

Stats: Merged Data	
Mean:	146.6 nm
Mode:	96.4 nm
SD:	69.2 nm
D10:	88.4 nm
D50:	126.2 nm
D90:	230.0 nm
Stats: Mean +/- Standard Error	
Mean:	146.8 +/- 2.6 nm
Mode:	97.9 +/- 2.0 nm
SD:	69.2 +/- 1.6 nm
D10:	88.8 +/- 1.6 nm
D50:	125.7 +/- 3.6 nm
D90:	228.0 +/- 5.9 nm
Concentration (Upgrade):	4.17e+08 +/- 1.69e+07 particles/ml
	98.0 +/- 2.8 particles/frame
	96.7 +/- 2.1 centres/frame

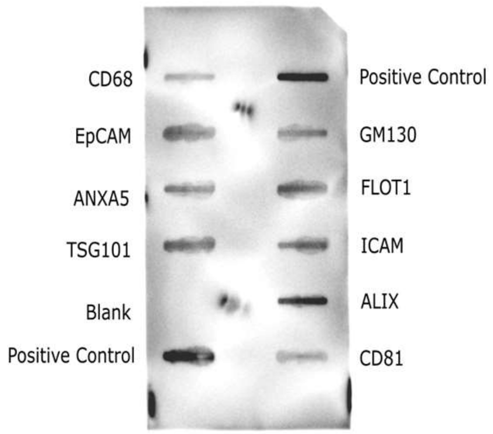

Supplementary Figure 1. Nanotracking Analysis and marker array of isolated MBV (N=3).

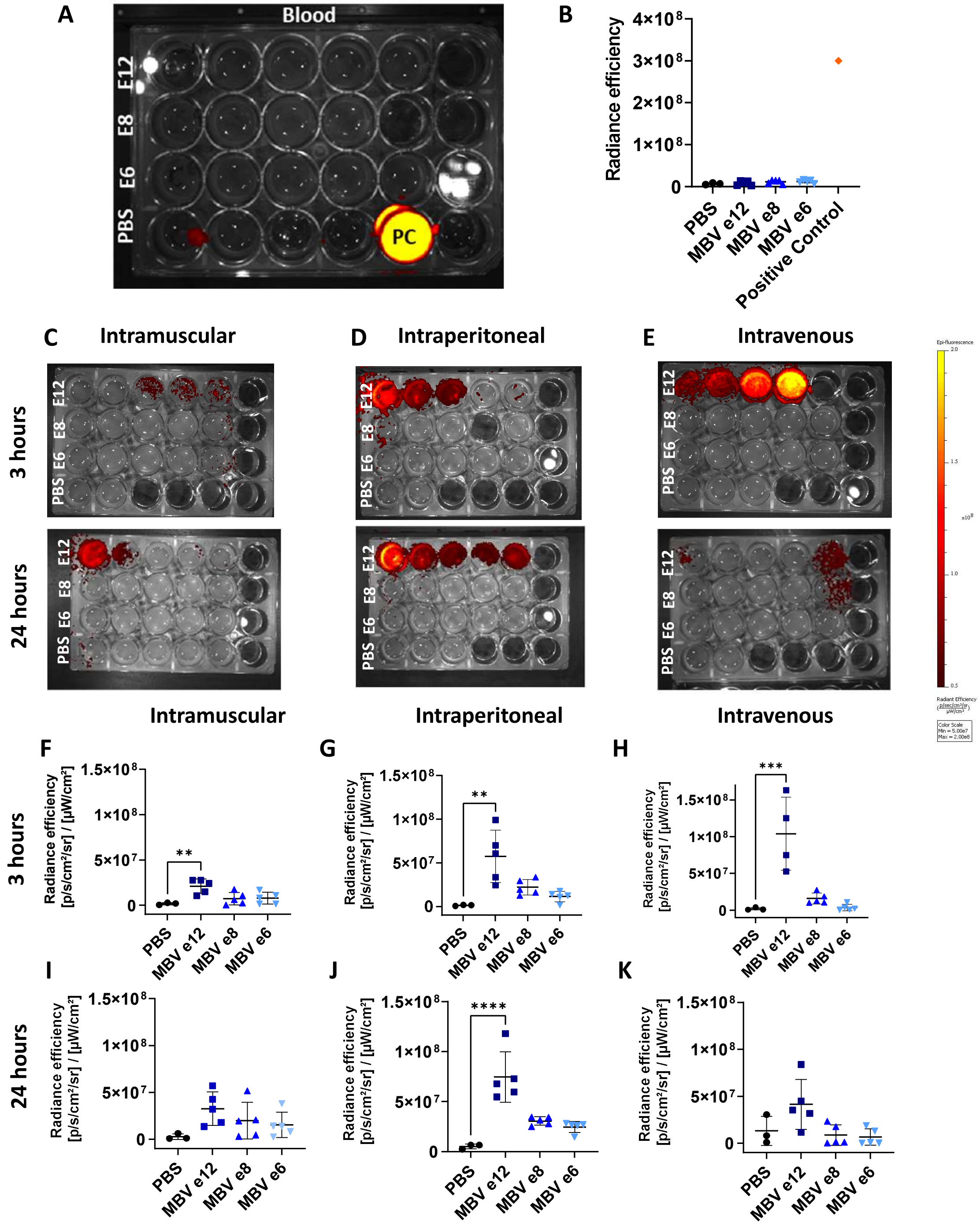

Supplementary Figure 2. IVIS imaging of bone marrow concentrates and blood. Leukocytes concentrated and isolated from blood did not show any signal, similarly to PBS negative control (**A)** and contrary to tagged MBV which presented a robust signal (**B).** On the other hand, bone marrow flushes from mice injected intramuscularly (**C**), intraperitoneally (**D**) and intravenously (**E**) showed detectable signal from fluorescently labelled MBV after 3 hours (**F-H**), although only in the highest dose of 10^{12} MBV/ml (10^{11} total MBV)**.** Only the highest dose and intraperitoneal delivery showed significant signal after 24 hours (**I-K**). (**, *** and **** indicate $p < 0.01$, 0.001 and 0.0001 (N=5) with PBS (N=3) respectively)

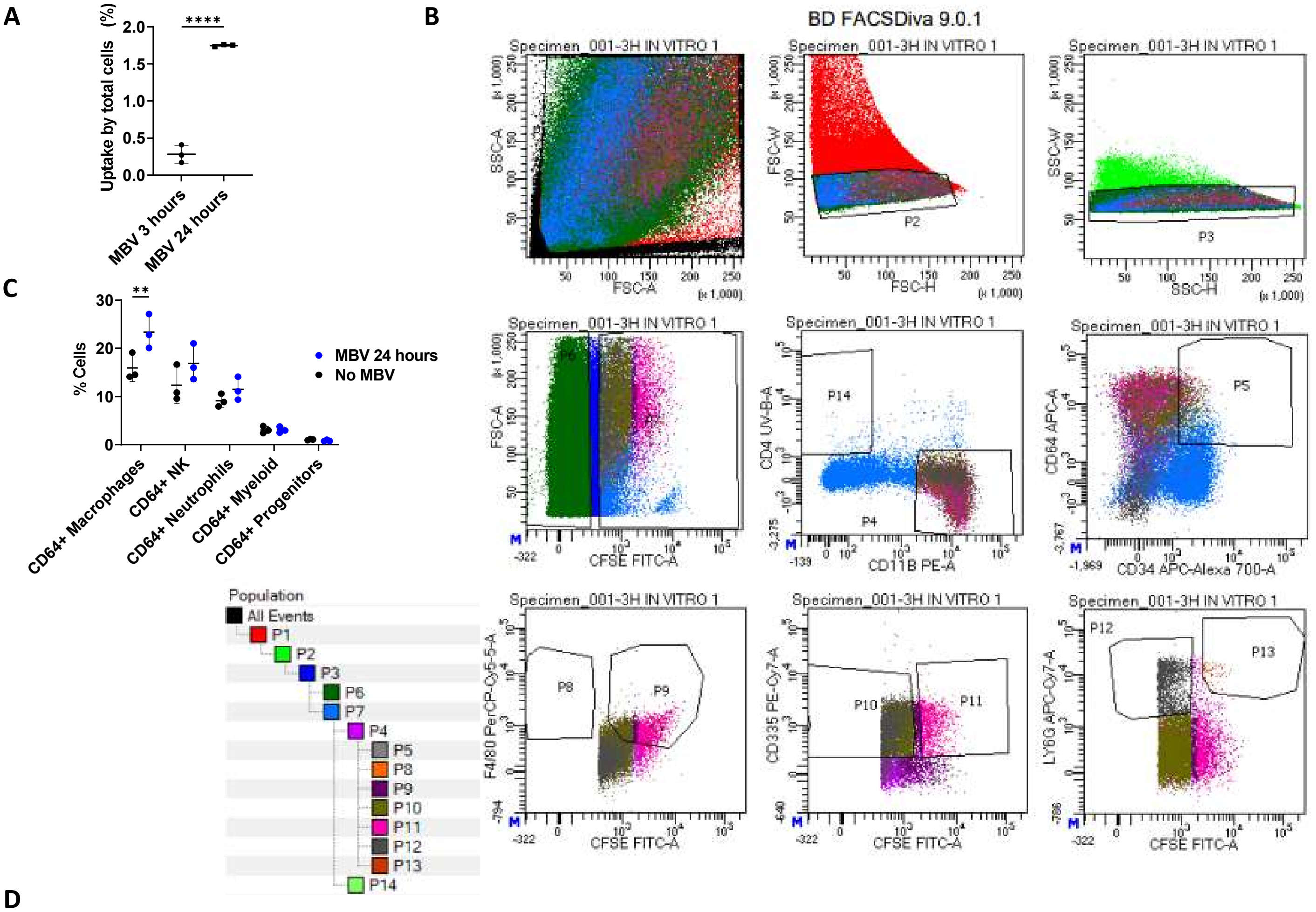

D

	MBV	Myeloid	Macrophages	Natural Killers	Neutrophils	Progenitors
			3 hours			
	**CFSE+ (P7)**	**CFSE+ CD11b+ (P4)**	**CFSE+ CD11b+/F480+ (P9)**	**CFSE+ CD11b+/CD335+ (P11)**	**CFSE+ CD11b+/LY6G+ (P13)**	**CFSE+/CD11b+/ CD64+/CD34+ (P5)**
Mean	0.28	68.17	11.76	22.00	43.83	10.08
SD	0.12	2.55	3.26	8.36	20.96	2.07
			24 hours			
	**CFSE+ (P7)**	**CFSE+ CD11b+ (P4)**	**CFSE+ CD11b+/F480+ (P9)**	**CFSE+ CD11b+/CD335+ (P11)**	**CFSE+ CD11b+/LY6G+ (P13)**	**CFSE+/CD11b+/ CD64+/CD34+ (P5)**
Mean	1.75	29.70	26.07	14.57	22.16	7.18
SD	0.02	2.21	1.00	8.72	10.98	0.14

Supplementary Figure 3. Flow cytometry analysis of bone marrow exposed to MBV *in vitro*. Total uptake analysis showed a low percentage of cells uptaking MBVs (**A**). Example of gating strategy (**B**). Total cell populations positive and negative for CD64 (**C**). Total quantification of cell populations after 3 and 24 hours expressed as mean and standard deviation (N=3) **(D)**.

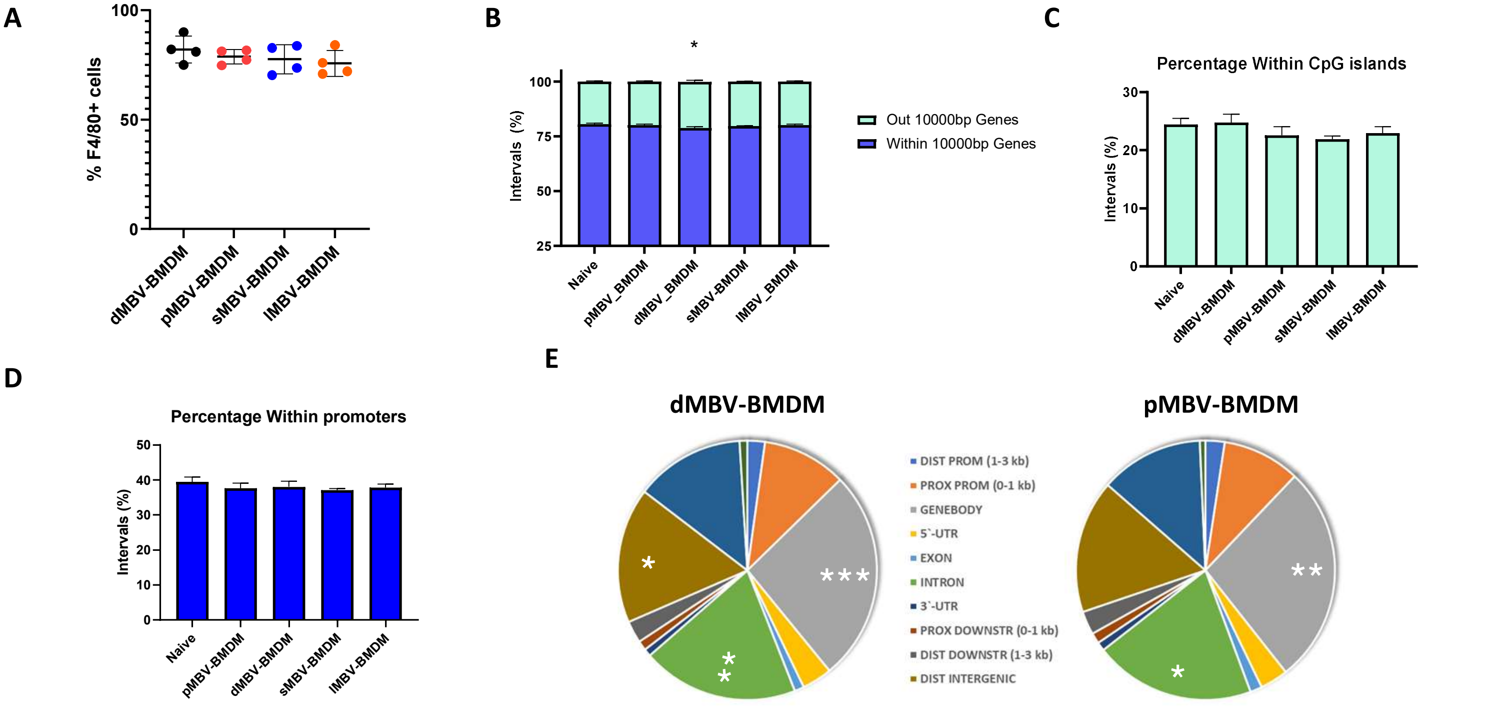

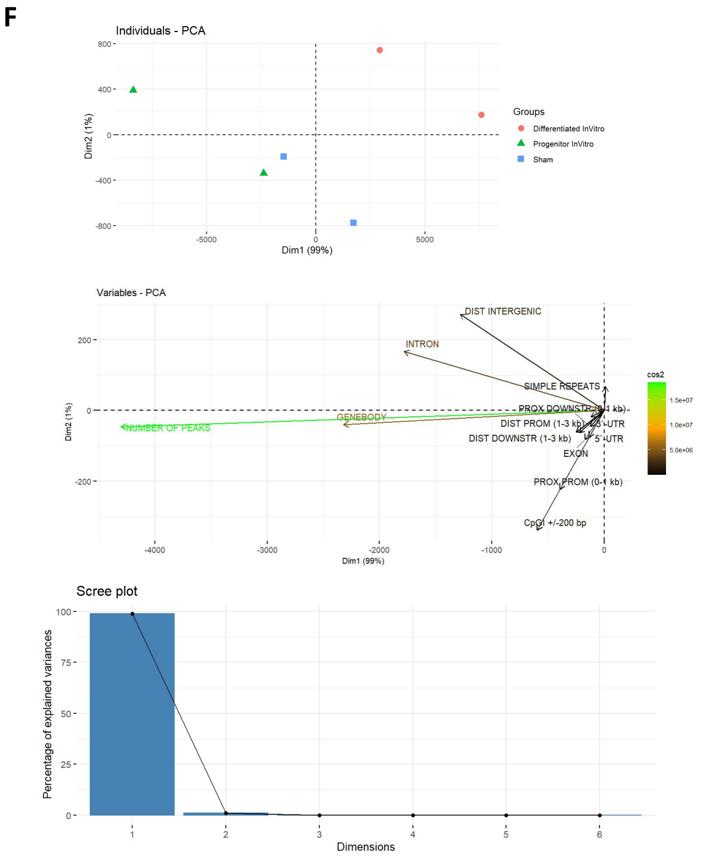

Supplementary Figure 4. ATAC-seq analysis of bone marrow derived macrophages (BMDM) exposed to MBV. All isolated groups of bone marrow differentiated macrophages showed most cells expressing F4/80, confirming their phenotype as macrophages (**A**). ATAC-seq analysis of bone marrow derived macrophages (BMDM) exposed to MBV *in vitro* showed similar distribution of peaks within genes (**B**), CpG islands (**C**) and promoters (**D**, * indicates p< 0.05 with native). When comparing macrophages exposed to MBV after differentiation (dMBV) and progenitor stage (pMBV), these groups showed similar distribution of peaks within the genome (**E,** *, ** and *** indicate *p* < 0.05, 0.01 and 0.001 compared to native genome peak distribution). Principal component analysis showed differences between samples, mainly explained by the variable number of peaks (**F**).

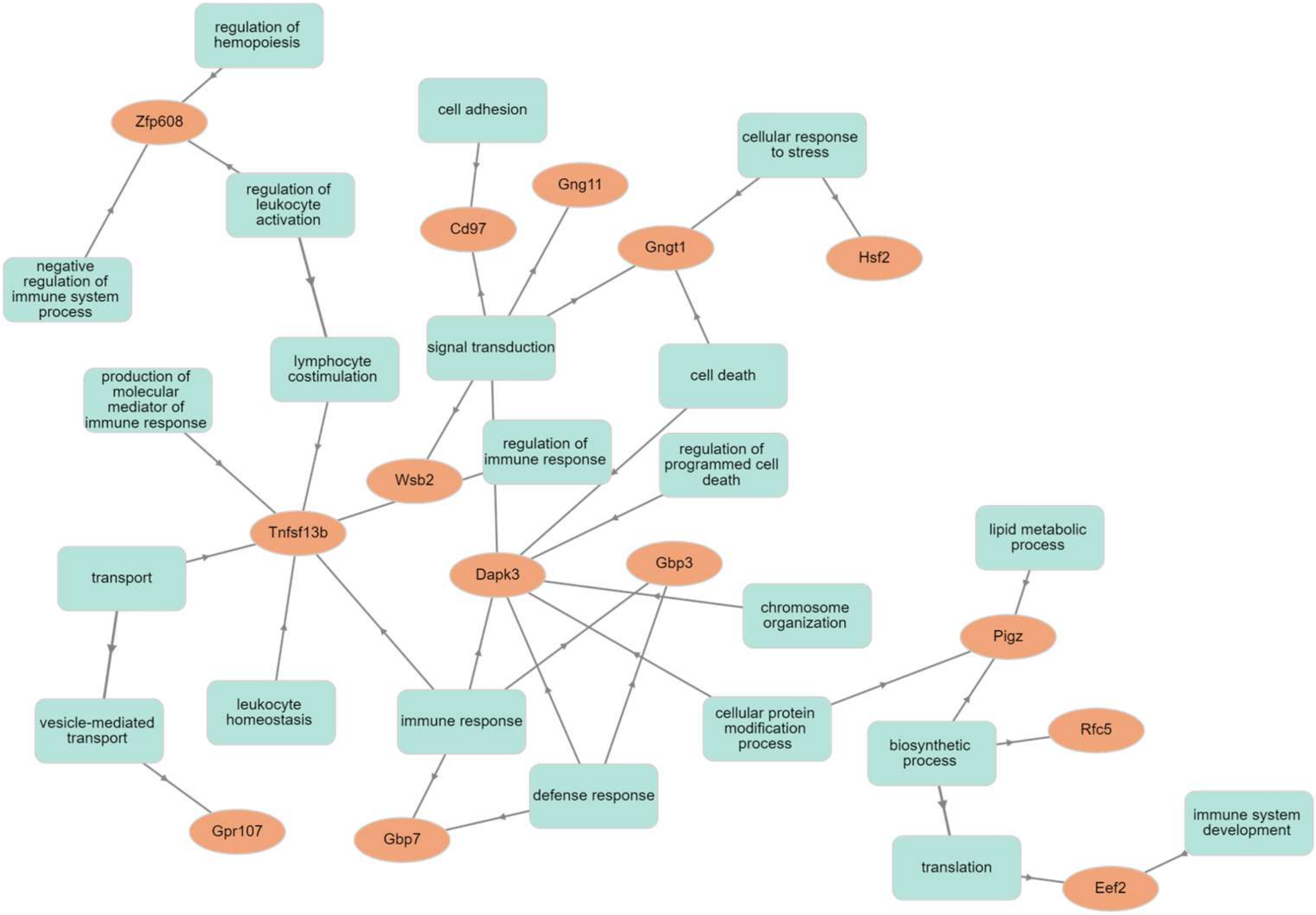

Supplementary Figure 5. Gene Ontology enrichment in immunological processes analysis was performed in epigenetically upregulated genes from differentiated macrophages (dMBV) exposed to MBV. A majority of epigenetically upregulated genes (circles) were related to functions (squares) such as leukocyte differentiation, adaptative, and innate immune response regulation.

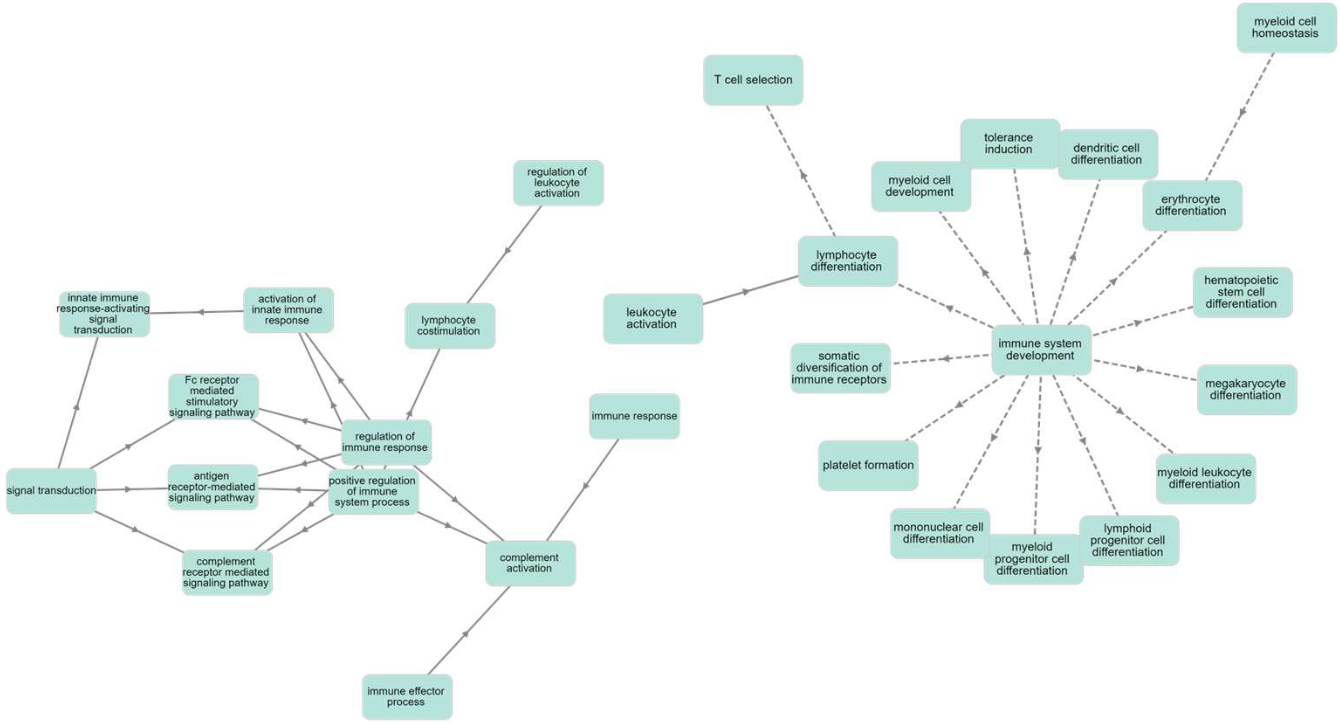

Supplementary Figure 6. Gene Ontology enrichment in immunological processes analysis was performed in epigenetically upregulated genes from macrophages exposed to MBV both in progenitor state (pMBV) and after differentiation (dMBV). Epigenetically upregulated (i.e. increased accessibility) genes in both pMBV and dMBV were related to leukocyte and monocyte-derived cells function regulation.

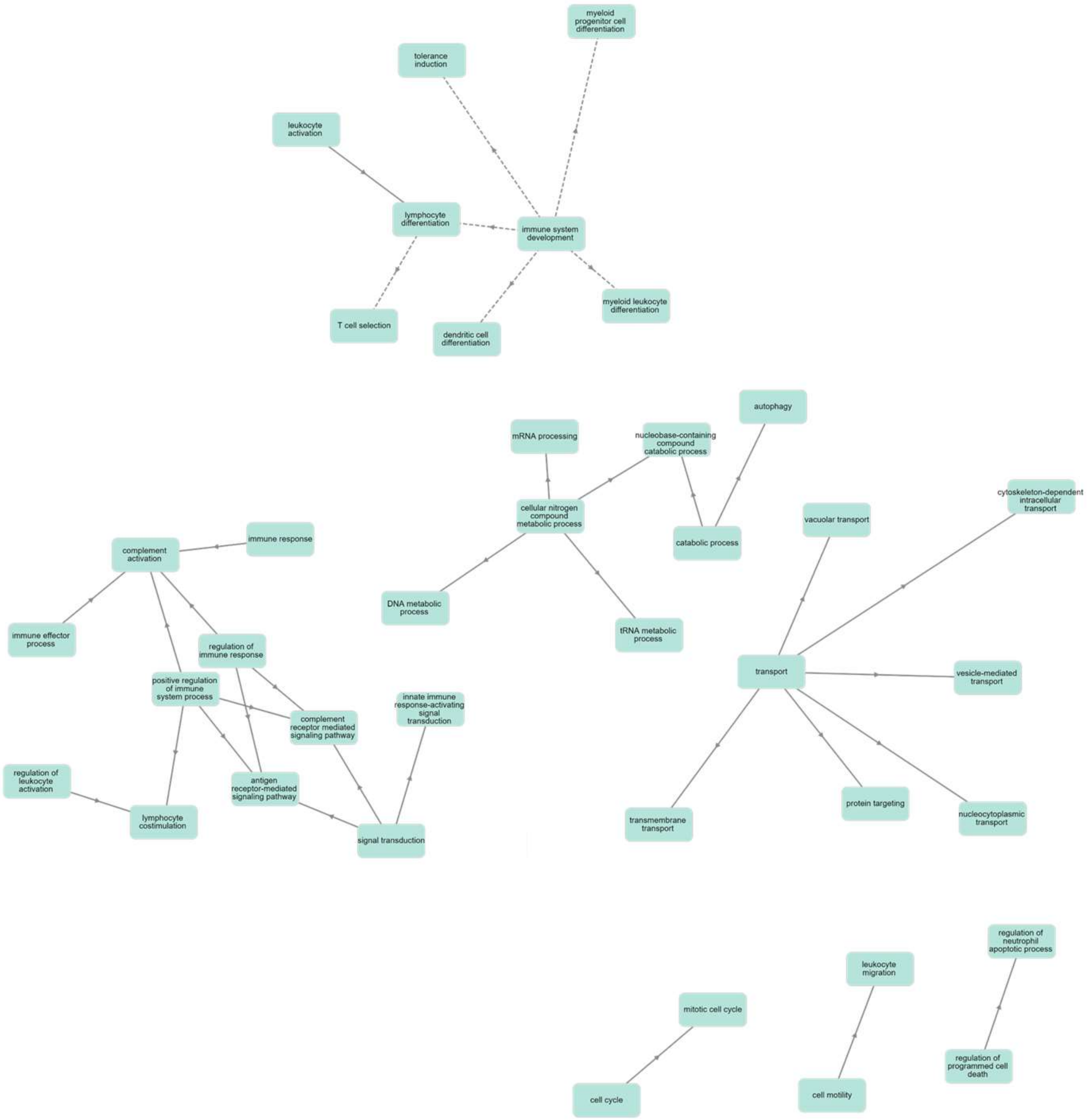

Supplementary Figure 7. Gene Ontology enrichment in immunological processes analysis of epigenetically upregulated genes from macrophages exposed to MBV in progenitor state (pMBV). Epigenetically upregulated genes (i.e. increased accessibility) in pMBV matched leukocyte and monocyte-derived cells function regulation.

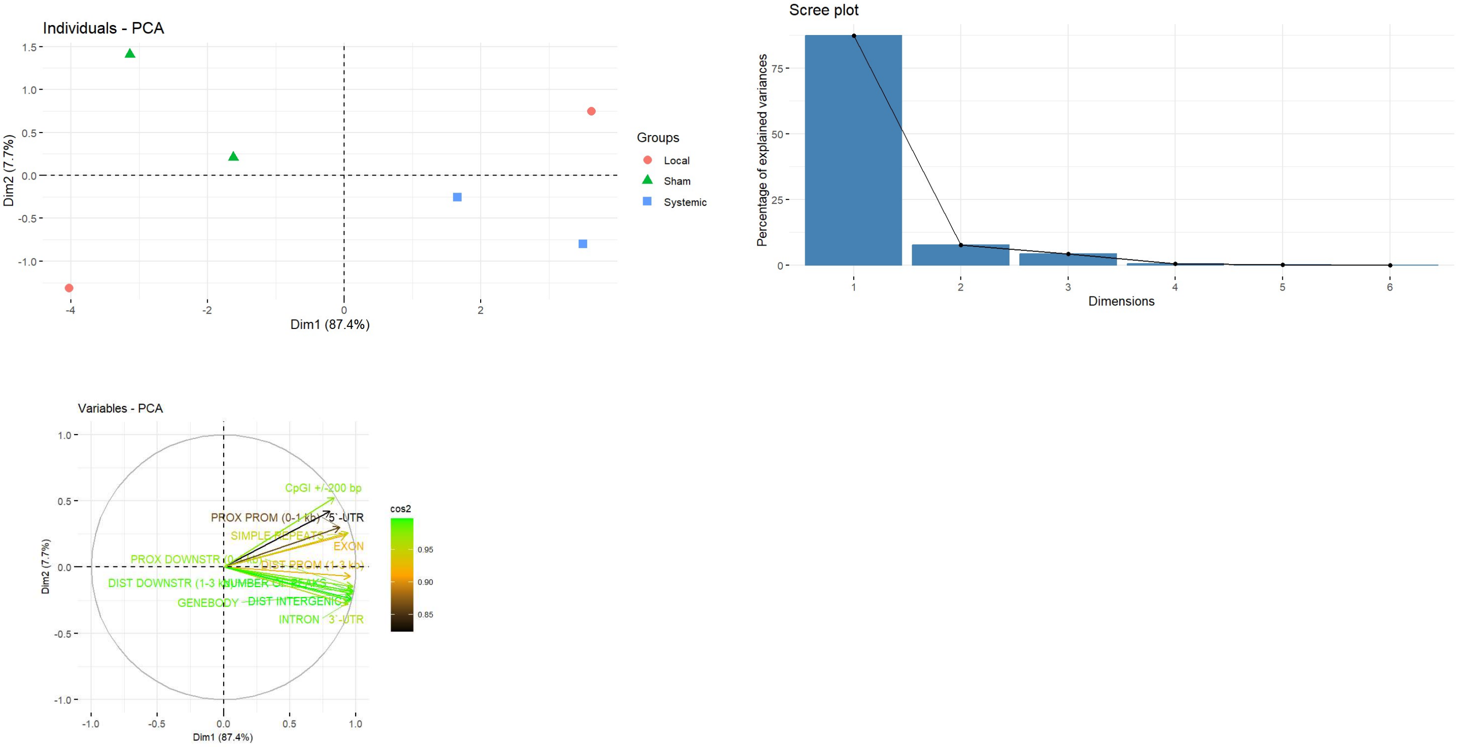

Supplementary Figure 8. ATAC-seq analysis of bone marrow derived macrophages (BMDM) exposed to MBV in vivo. PCA analysis of BMDM exposed to MBV *in vivo* after local (lMBV) and systemic (sMBV) delivery, just showed differences between lMBV BMDM and naïve BMDM.

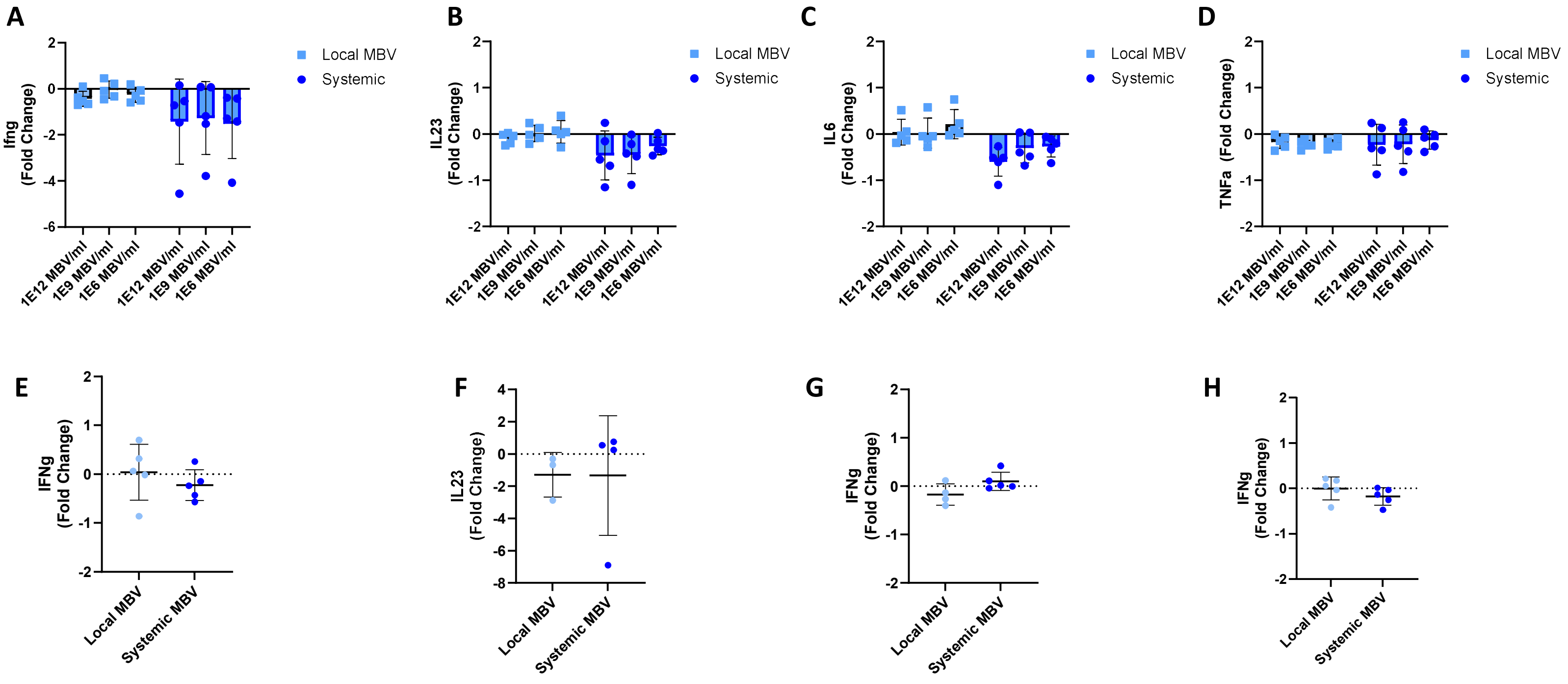

Supplementary Figure 9. RT-qPCR of inflammatory cytokines in bone marrow derived macrophages (BMDM) isolated from mice exposed to MBV *in vivo* at different doses and regimes. BMDM derived from mice injected with one dose of MBV at 10^{12}, 10^{9} and 10^{6} MBV/ml both locally and systemically and exposed to LPS *in vitro* (**A-D**) showed no differences in IFN-γ (**A**), IL23 (**B**), IL6 (**C**) nor TNFα (**D**) expression fold change respect to naïve macrophages. Similarly, findings were observed after repeated dosing of 10^{8} total MBV both systemically and locally (**E-H**). (N=5, data as average and standard deviation

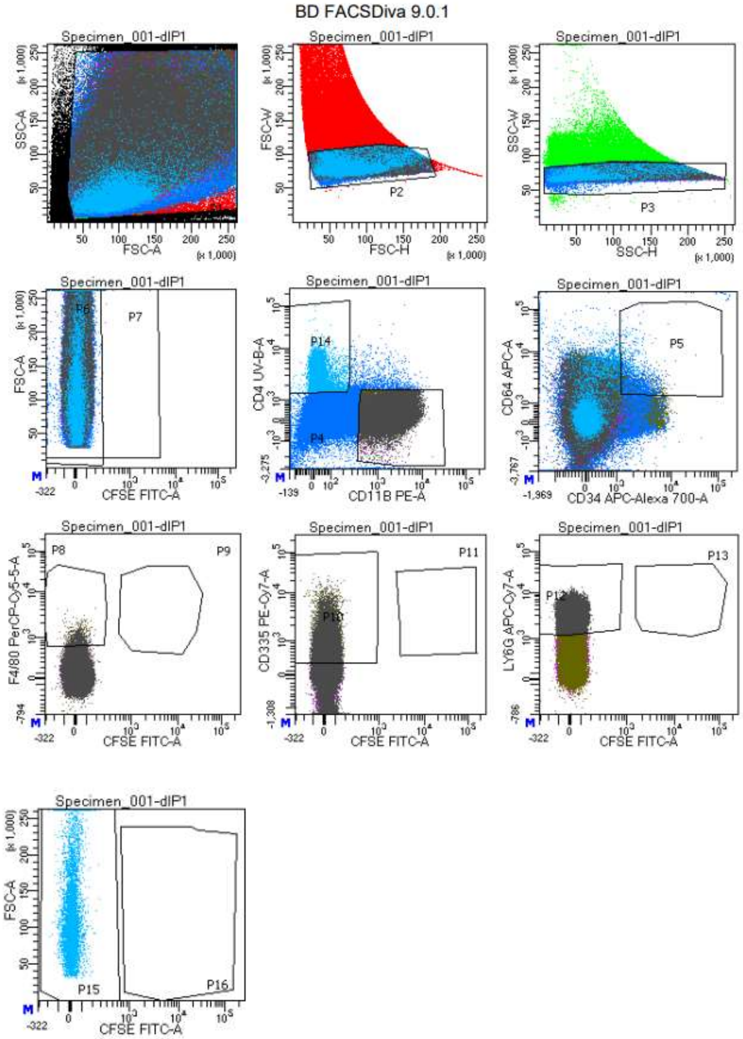

Tube: dIP1

Population	#Events	%Parent	%Total
All Events	4,049,928	####	100.0
P1	3,011,609	74.4	74.4
P2	2,827,403	93.9	69.8
P3	2,794,025	98.8	69.0
P6	2,794,013	100.0	69.0
P7	2,794,025	100.0	69.0
P4	590,549	21.1	14.6
P5	210	0.0	0.0
P8	996	0.2	0.0
P9	0	0.0	0.0
P10	149,326	25.3	3.7
P11	0	0.0	0.0
P12	425,731	72.1	10.5
P13	0	0.0	0.0
P14	12,557	0.4	0.3
P15	12,557	100.0	0.3
P16	0	0.0	0.0

Supplementary Figure 10. Flow cytometry analysis of bone marrow exposed to MBV *in vivo*. Fluorescence readings of cells positive for CFSE retrieved no positive results, as depicted by the count of population P6 (negative for CFSE) and P7 (negative + positive for CFSE).

Upregulated

dMBV_BMDM Motif	P-value	log P-value	% Targets	% Background
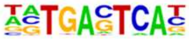	1e-392	-9.036e+02	42.82%	8.06%
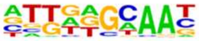	1e-145	-3.340e+02	37.40%	14.30%
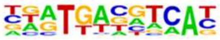	1e-113	-2.623e+02	37.05%	16.12%
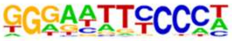	1e-113	-2.609e+02	17.51%	4.19%
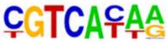	1e-95	-2.193e+02	45.83%	24.57%

pMBV_BMDM Motif	P-value	log P-pvalue	% Targets	% Background
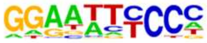	1e-104	-2.415e+02	31.89%	3.72%
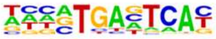	1e-32	-7.386e+01	25.85%	8.40%
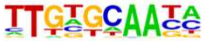	1e-26	-6.101e+01	24.34%	8.63%
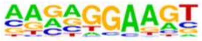	1e-22	-5.140e+01	42.45%	23.09%
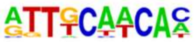	1e-18	-4.298e+01	27.17%	12.61%

Downregualted

dMBV_BMDM Motif	P-value	log P-pvalue	% Targets	% Background
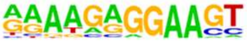	1e-206	-4.744e+02	21.63%	4.12%
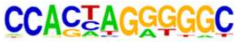	1e-44	-1.028e+02	4.31%	0.71%
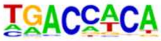	1e-25	-5.965e+01	18.34%	10.92%
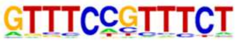	1e-24	-5.651e+01	4.01%	1.11%
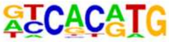	1e-21	-4.965e+01	22.01%	14.55%

pMBV_BMDM Motif	P-value	log P-pvalue	% of Targets	% Background
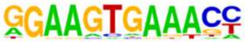	1e-24	-5.642e+01	18.18%	1.95%
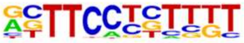	1e-20	-4.735e+01	24.40%	5.00%
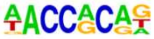	1e-17	-4.019e+01	37.80%	13.70%

Supplementary Table 1. Top ranked epigenetically upregulated and downregulated motif sequences in macrophages exposed to MBV both in progenitor state (pMBV) and after differentiation (dMBV). HOMER analysis of motifs in upregulated and downregulated sequences from ATAC-seq analysis macrophages exposed to MBV both in progenitor state (pMBV) and after differentiation (dMBV) were ranked in function of lower p-value, higher % match with targets and lower % match with background, by this order of importance.

Supplementary data. Gating and FMO data and strategy used in flow cytometry.

BD FACSDiva 9.0.1

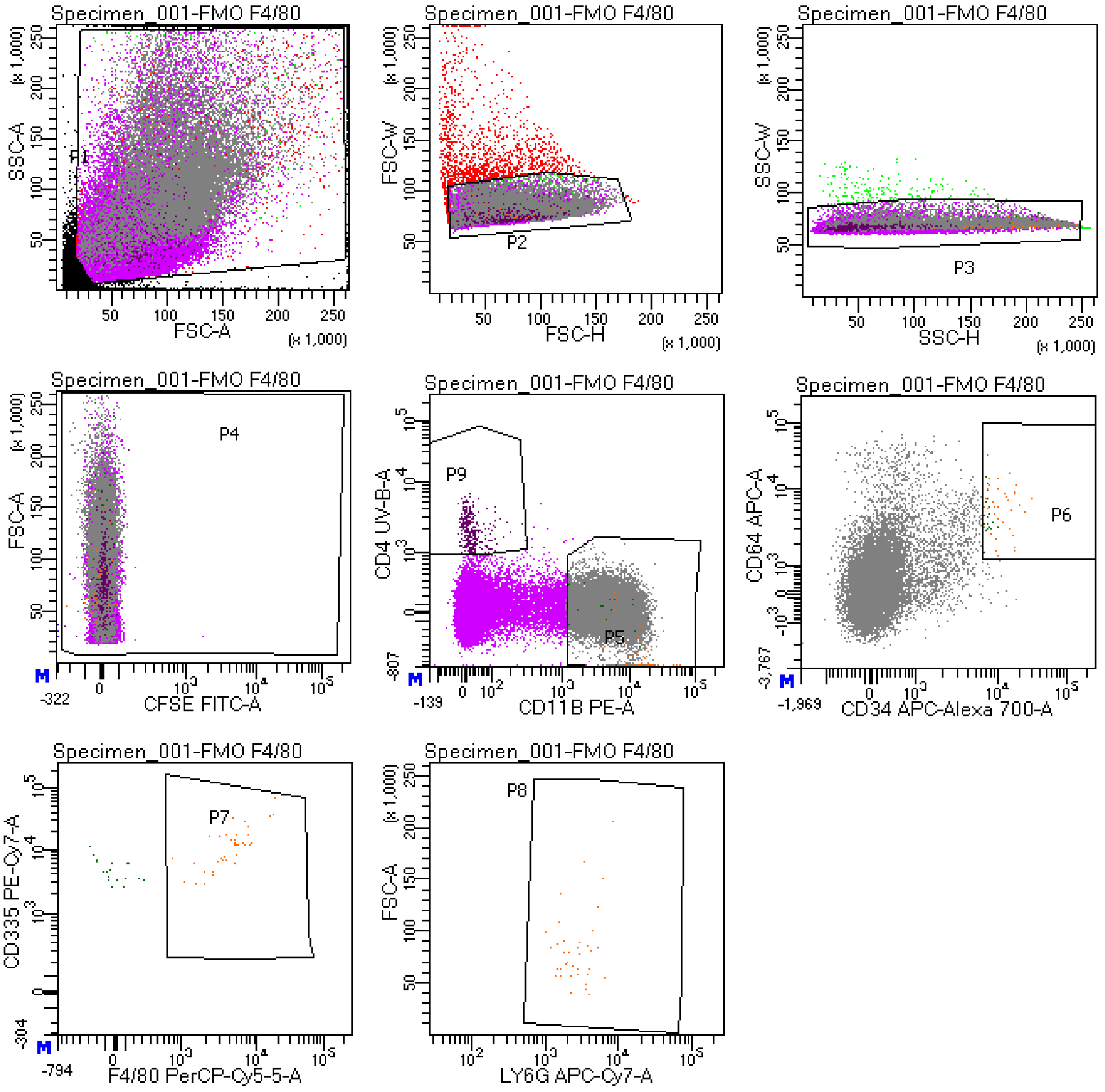

Tube: FMO F4/80

Population	#Events	%Parent	%Total
All Events	100,000	####	100.0
P1	84,633	84.6	84.6
P2	82,901	98.0	82.9
P3	82,716	99.8	82.7
P4	82,713	100.0	82.7
P5	15,159	18.3	15.2
P6	55	0.4	0.1
P7	36	65.5	0.0
P8	36	100.0	0.0
P9	282	0.3	0.3

BD FACSDiva 9.0.1

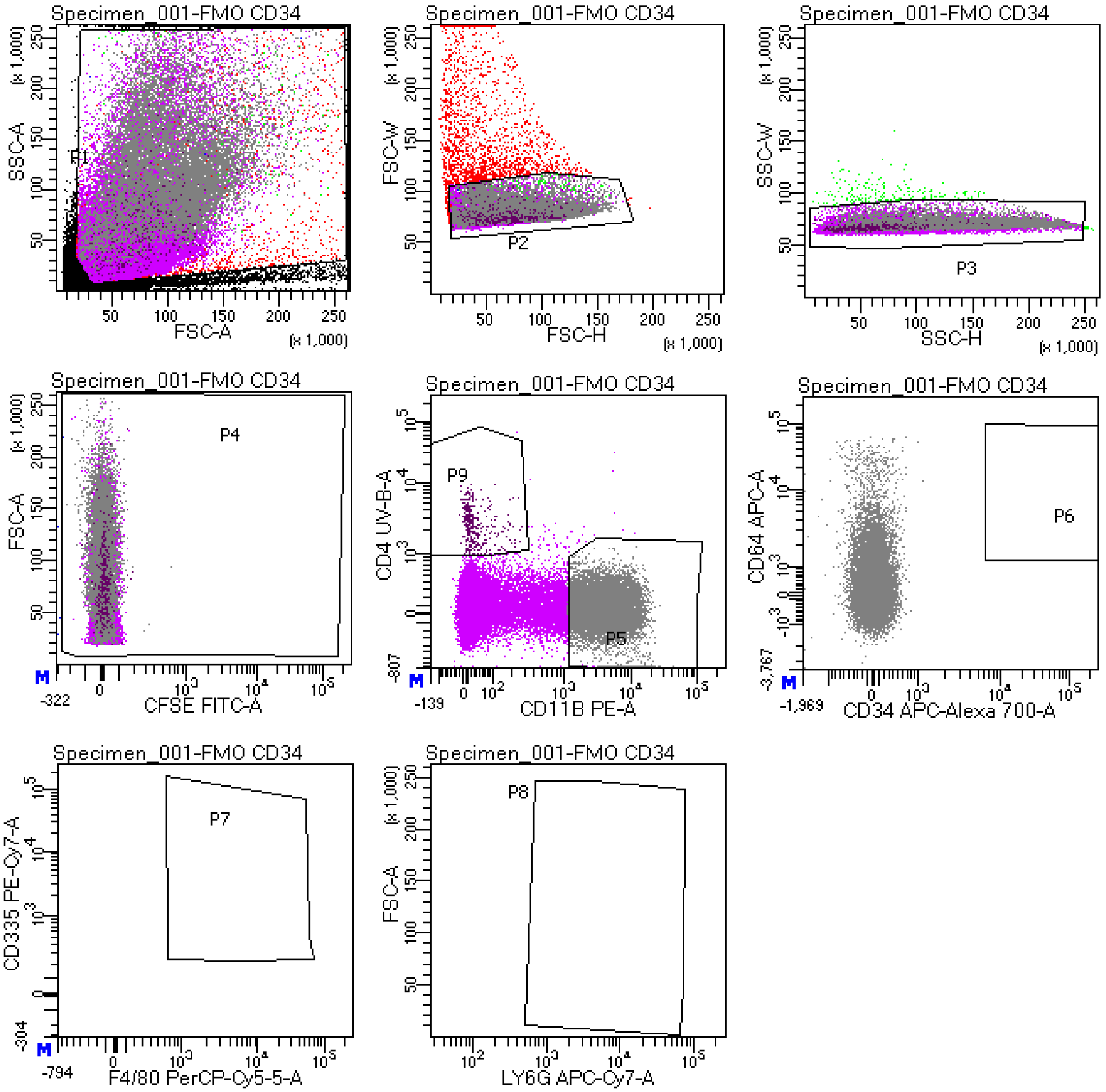

Tube: FMO CD34

Population	#Events	%Parent	%Total
All Events	100,000	####	100.0
P1	74,969	75.0	75.0
P2	72,611	96.9	72.6
P3	72,417	99.7	72.4
P4	72,411	100.0	72.4
P5	13,649	18.8	13.6
P6	0	0.0	0.0
P7	0	####	0.0
P8	0	####	0.0
P9	330	0.5	0.3

BD FACSDiva 9.0.1

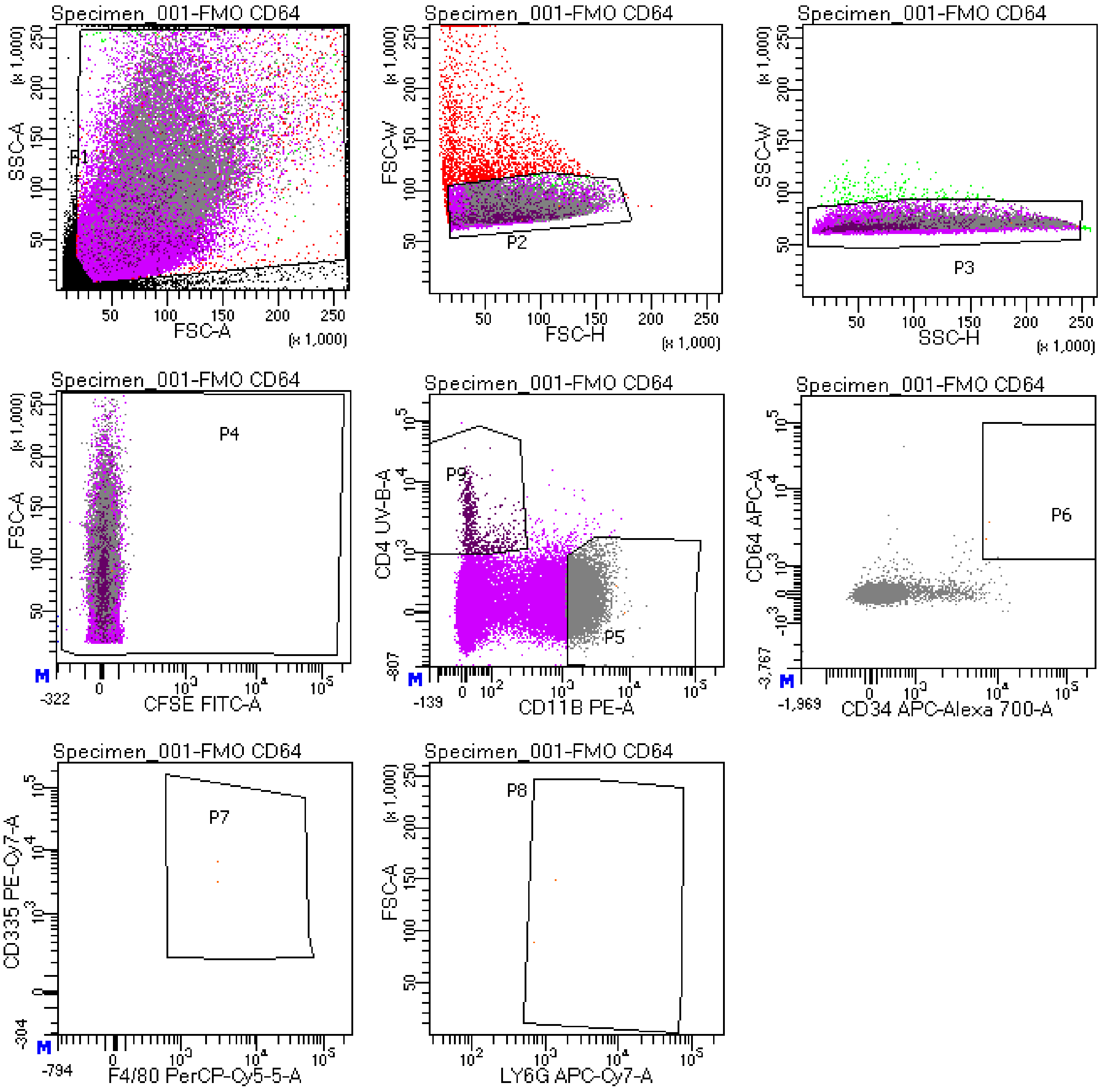

Tube: FMO CD64

Population	#Events	%Parent	%Total
All Events	100,000	####	100.0
P1	79,475	79.5	79.5
P2	76,989	96.9	77.0
P3	76,763	99.7	76.8
P4	76,759	100.0	76.8
P5	7,480	9.7	7.5
P6	2	0.0	0.0
P7	2	100.0	0.0
P8	2	100.0	0.0
P9	696	0.9	0.7

BD FACSDiva 9.0.1

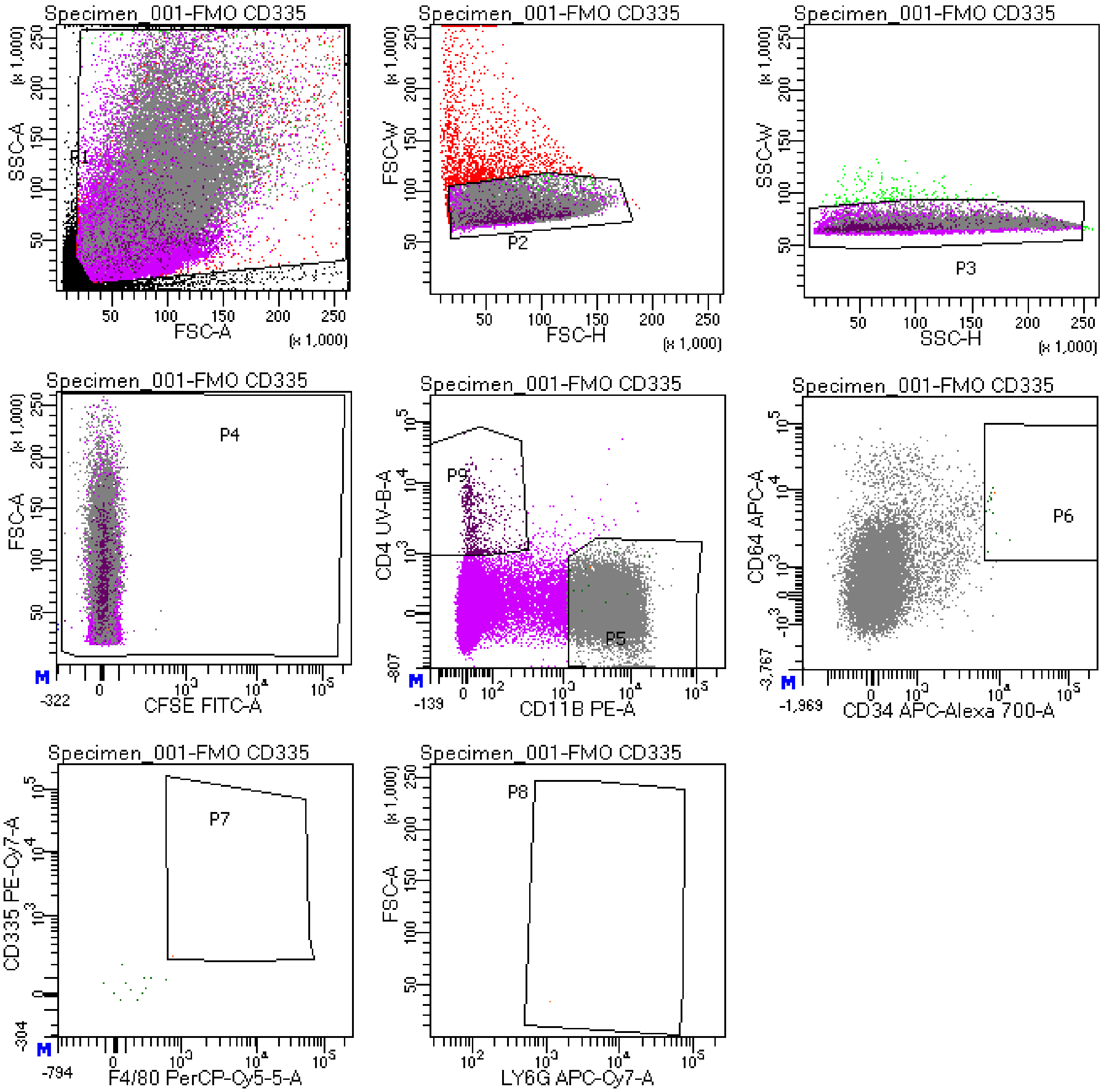

Tube: FMO CD335

Population	#Events	%Parent	%Total
All Events	100,000	####	100.0
P1	81,114	81.1	81.1
P2	78,972	97.4	79.0
P3	78,788	99.8	78.8
P4	78,786	100.0	78.8
P5	15,544	19.7	15.5
P6	13	0.1	0.0
P7	1	7.7	0.0
P8	1	100.0	0.0
P9	585	0.7	0.6

BD FACSDiva 9.0.1

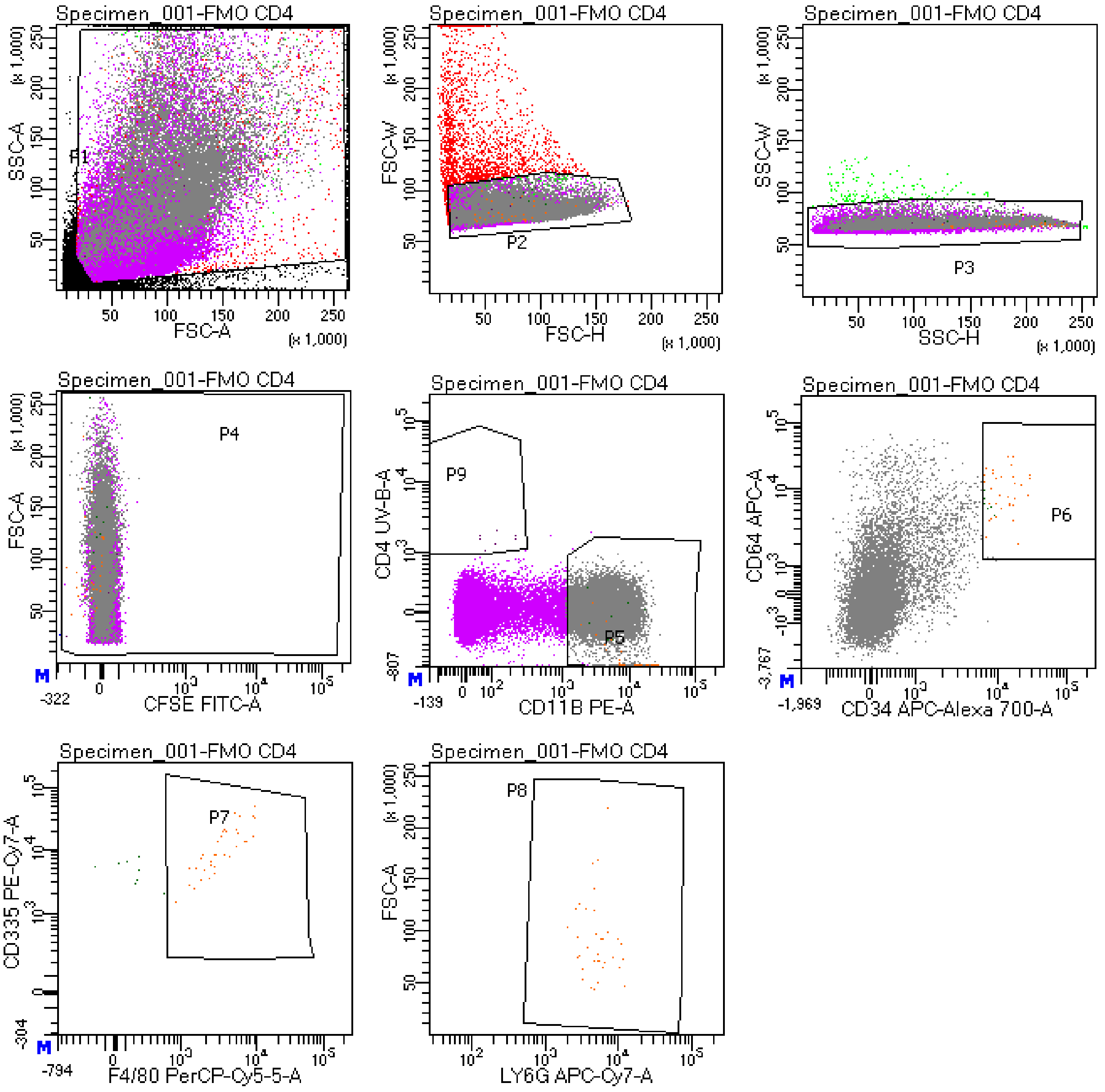

Tube: FMO CD4

Population	#Events	%Parent	%Total
All Events	100,000	####	100.0
P1	69,442	69.4	69.4
P2	67,168	96.7	67.2
P3	67,017	99.8	67.0
P4	67,015	100.0	67.0
P5	11,645	17.4	11.6
P6	42	0.4	0.0
P7	34	81.0	0.0
P8	34	100.0	0.0
P9	7	0.0	0.0

BD FACSDiva 9.0.1

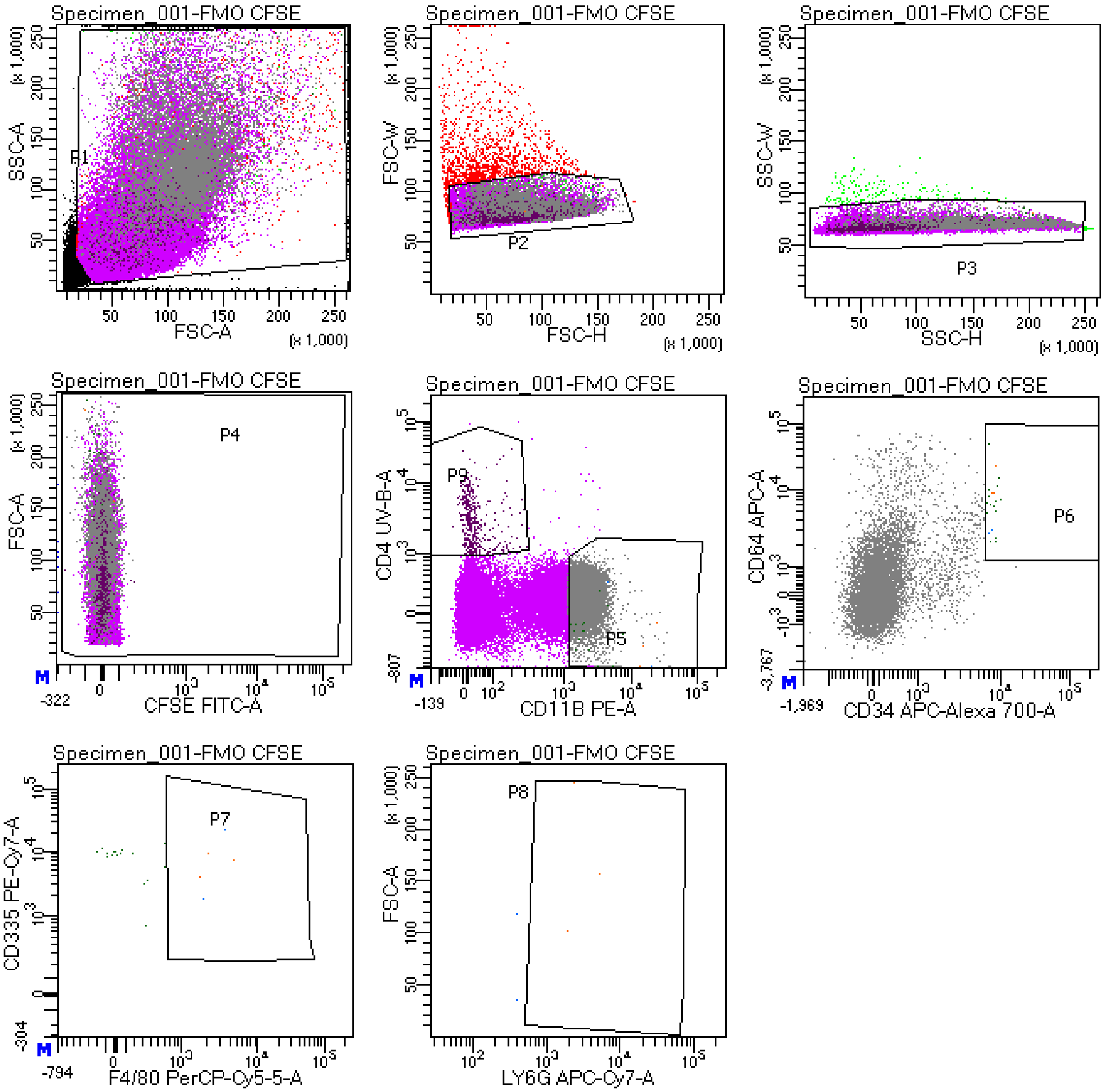

Tube: FMO CFSE

Population	#Events	%Parent	%Total
All Events	100,000	####	100.0
P1	85,379	85.4	85.4
P2	83,646	98.0	83.6
P3	83,463	99.8	83.5
P4	83,453	100.0	83.5
P5	8,993	10.8	9.0
P6	21	0.2	0.0
P7	5	23.8	0.0
P8	3	60.0	0.0
P9	476	0.6	0.5

BD FACSDiva 9.0.1

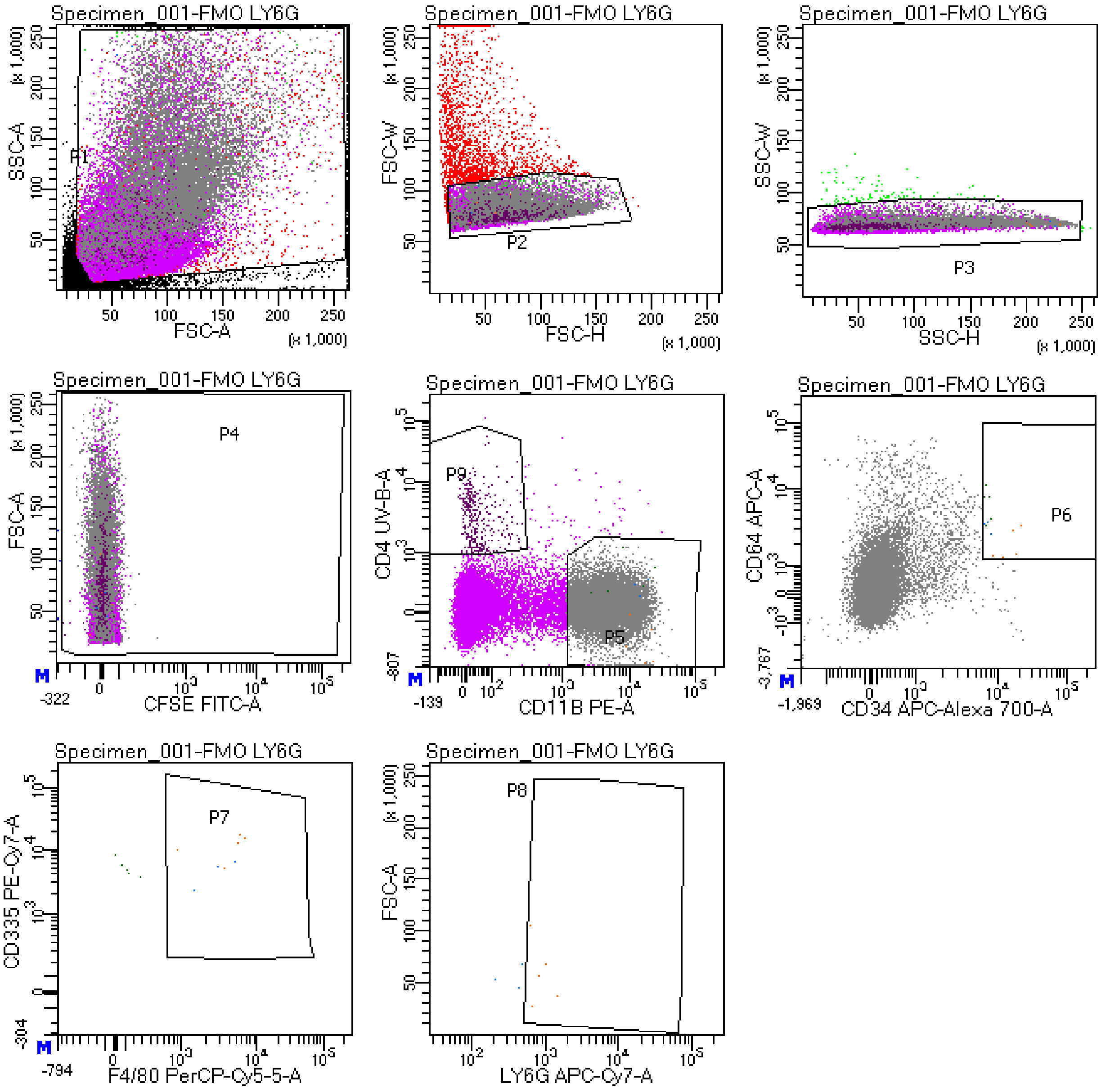

Tube: FMO LY6G

Population	#Events	%Parent	%Total
All Events	100,000	####	100.0
P1	55,778	55.8	55.8
P2	53,453	95.8	53.5
P3	53,347	99.8	53.3
P4	53,343	100.0	53.3
P5	11,161	20.9	11.2
P6	13	0.1	0.0
P7	8	61.5	0.0
P8	5	62.5	0.0
P9	352	0.7	0.4

Printed on: Fri Apr 21, 2023 02:48:59 PM EDT

BD FACSDiva 9.0.1

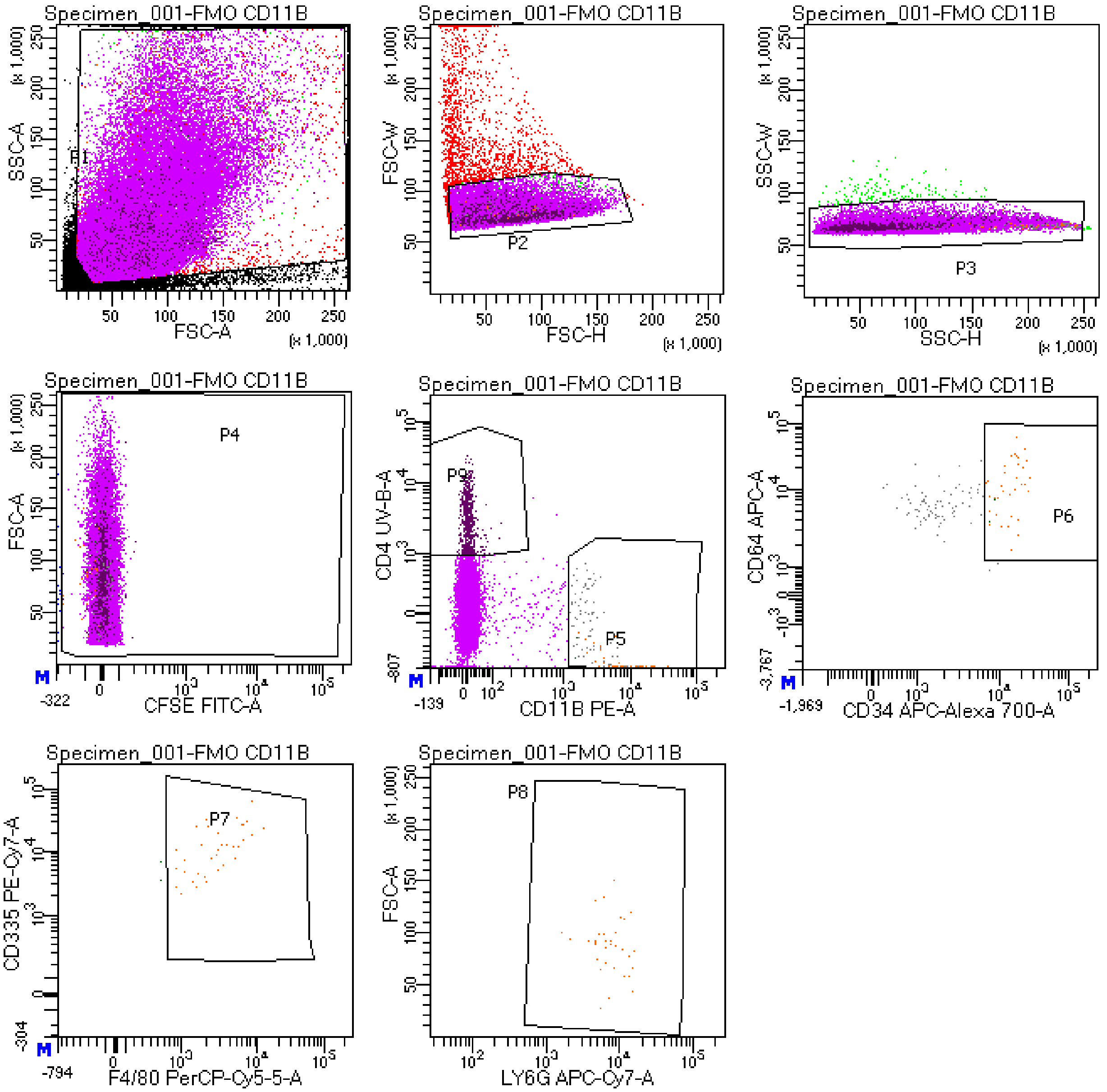

Tube: FMO CD11B

Population	#Events	%Parent	%Total
All Events	100,000	####	100.0
P1	76,293	76.3	76.3
P2	74,151	97.2	74.2
P3	73,994	99.8	74.0
P4	73,982	100.0	74.0
P5	113	0.2	0.1
P6	36	31.9	0.0
P7	34	94.4	0.0
P8	34	100.0	0.0
P9	640	0.9	0.6

BD FACSDiva 9.0.1

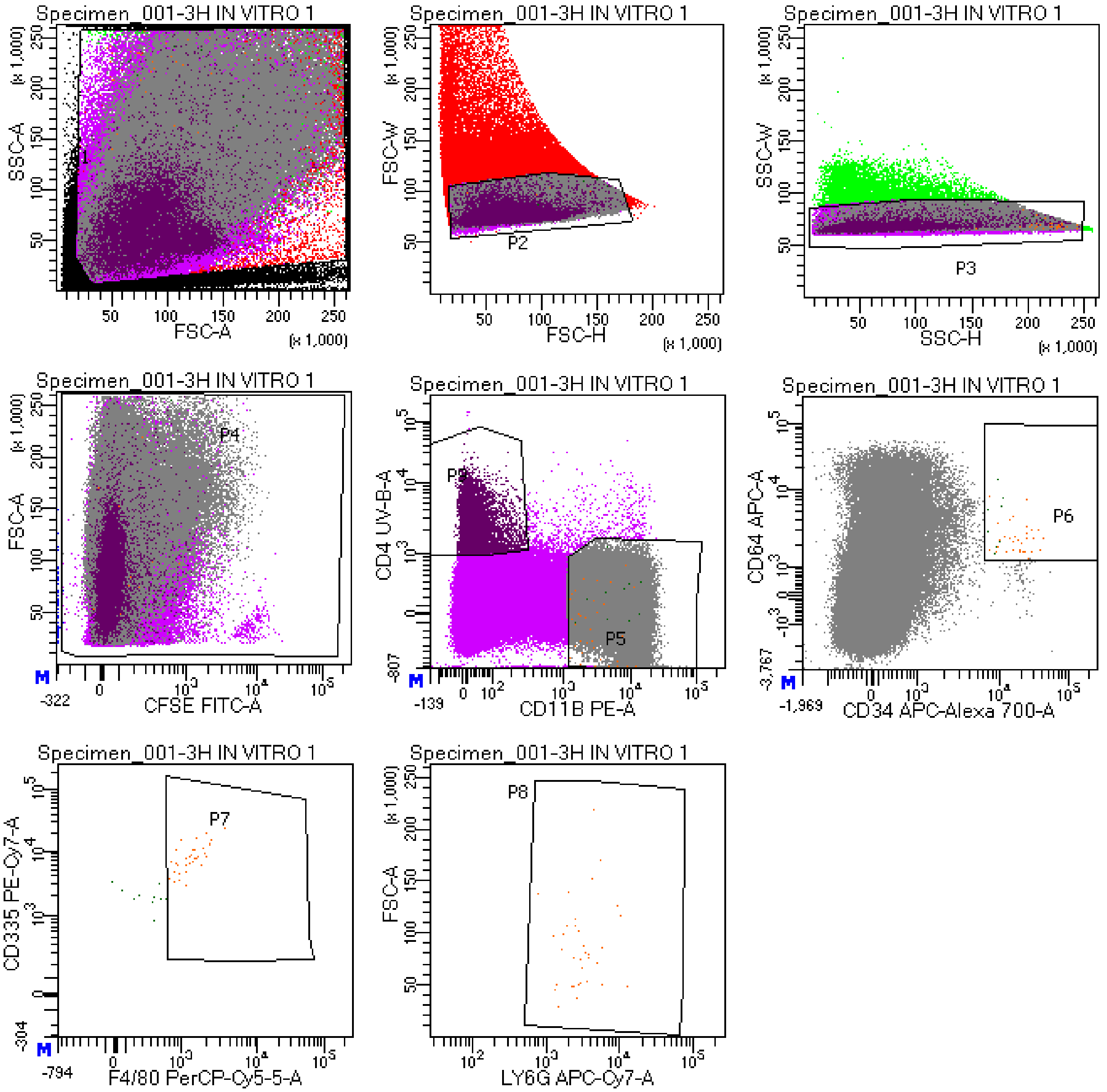

Tube: 3H IN VITRO 1

Population	#Events	%Parent	%Total
All Events	1,918,411	####	100.0
P1	1,649,784	86.0	86.0
P2	1,616,135	98.0	84.2
P3	1,609,893	99.6	83.9
P4	1,609,835	100.0	83.9
P5	251,158	15.6	13.1
P6	44	0.0	0.0
P7	34	77.3	0.0
P8	34	100.0	0.0
P9	10,030	0.6	0.5

BD FACSDiva 9.0.1

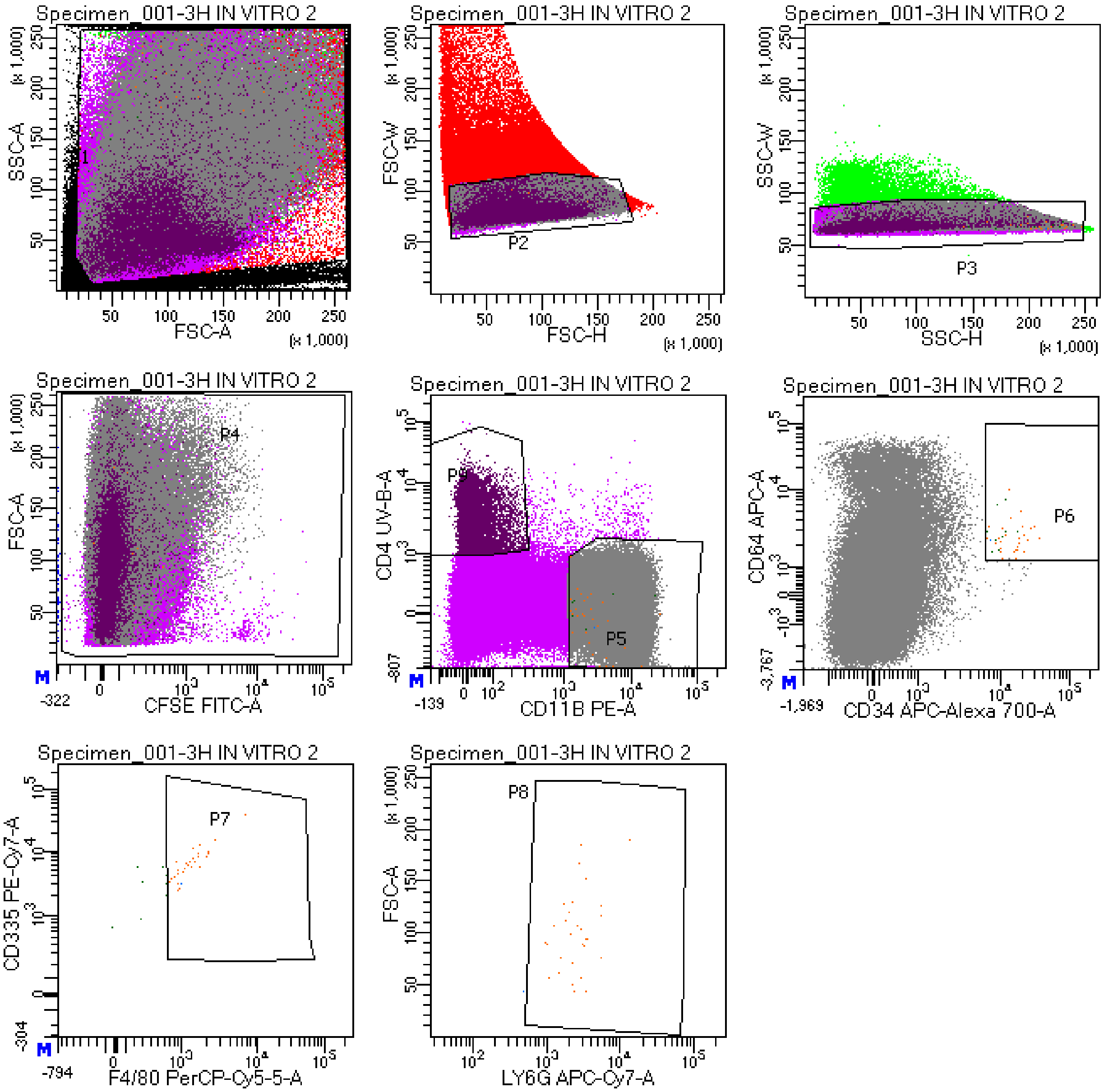

Tube: 3H IN VITRO 2

Population	#Events	%Parent	%Total
All Events	1,722,966	####	100.0
P1	1,460,093	84.7	84.7
P2	1,414,638	96.9	82.1
P3	1,407,972	99.5	81.7
P4	1,407,918	100.0	81.7
P5	228,228	16.2	13.2
P6	39	0.0	0.0
P7	30	76.9	0.0
P8	29	96.7	0.0
P9	9,829	0.7	0.6

BD FACSDiva 9.0.1

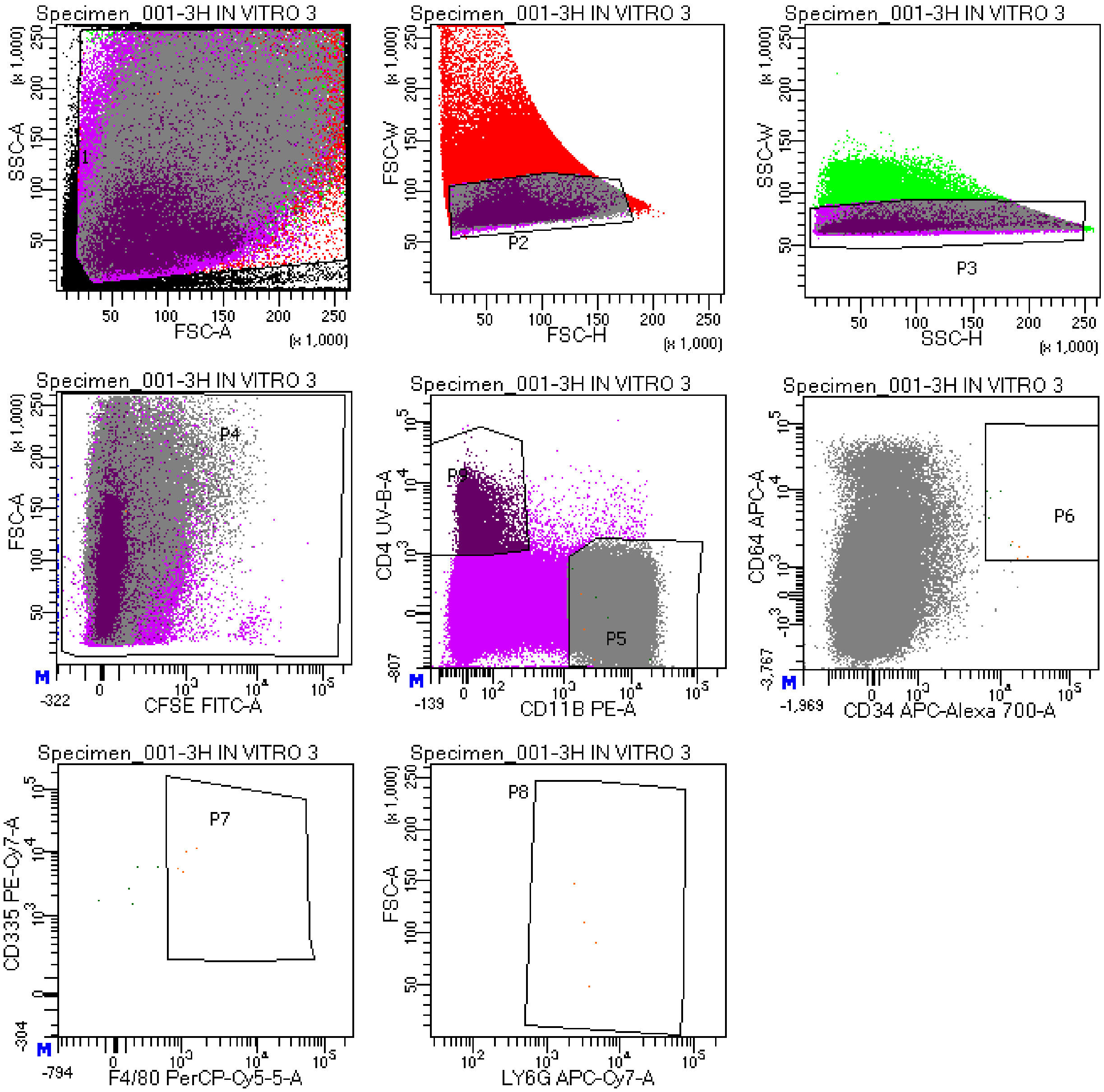

Tube: 3H IN VITRO 3

Population	#Events	%Parent	%Total
All Events	2,000,000	####	100.0
P1	1,791,385	89.6	89.6
P2	1,736,865	97.0	86.8
P3	1,722,927	99.2	86.1
P4	1,722,871	100.0	86.1
P5	284,635	16.5	14.2
P6	9	0.0	0.0
P7	4	44.4	0.0
P8	4	100.0	0.0
P9	11,579	0.7	0.6

BD FACSDiva 9.0.1

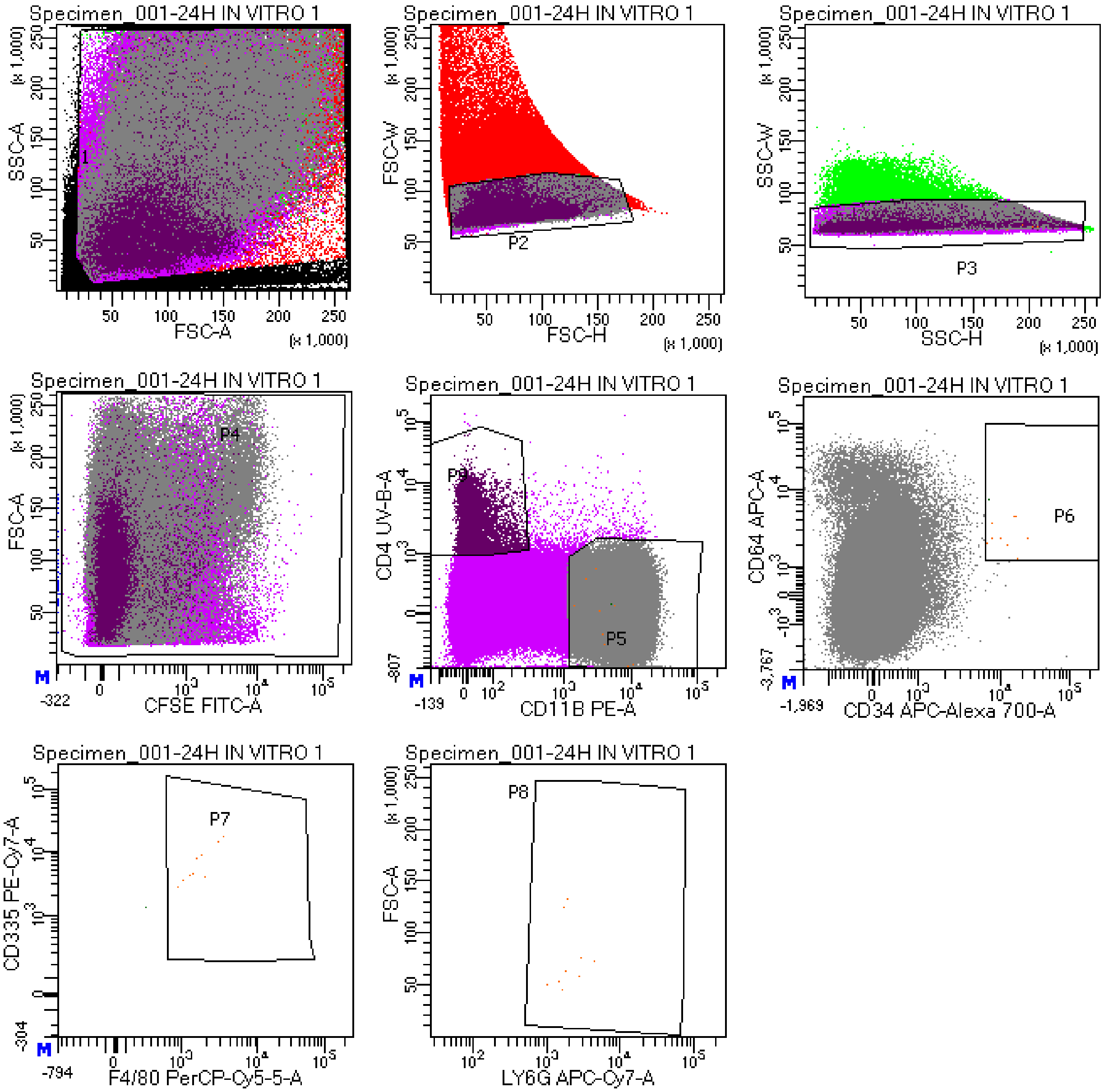

Tube: 24H IN VITRO 1

Population	#Events	%Parent	%Total
All Events	2,000,000	####	100.0
P1	1,733,034	86.7	86.7
P2	1,669,630	96.3	83.5
P3	1,659,606	99.4	83.0
P4	1,659,560	100.0	83.0
P5	277,485	16.7	13.9
P6	10	0.0	0.0
P7	9	90.0	0.0
P8	9	100.0	0.0
P9	9,959	0.6	0.5

BD FACSDiva 9.0.1

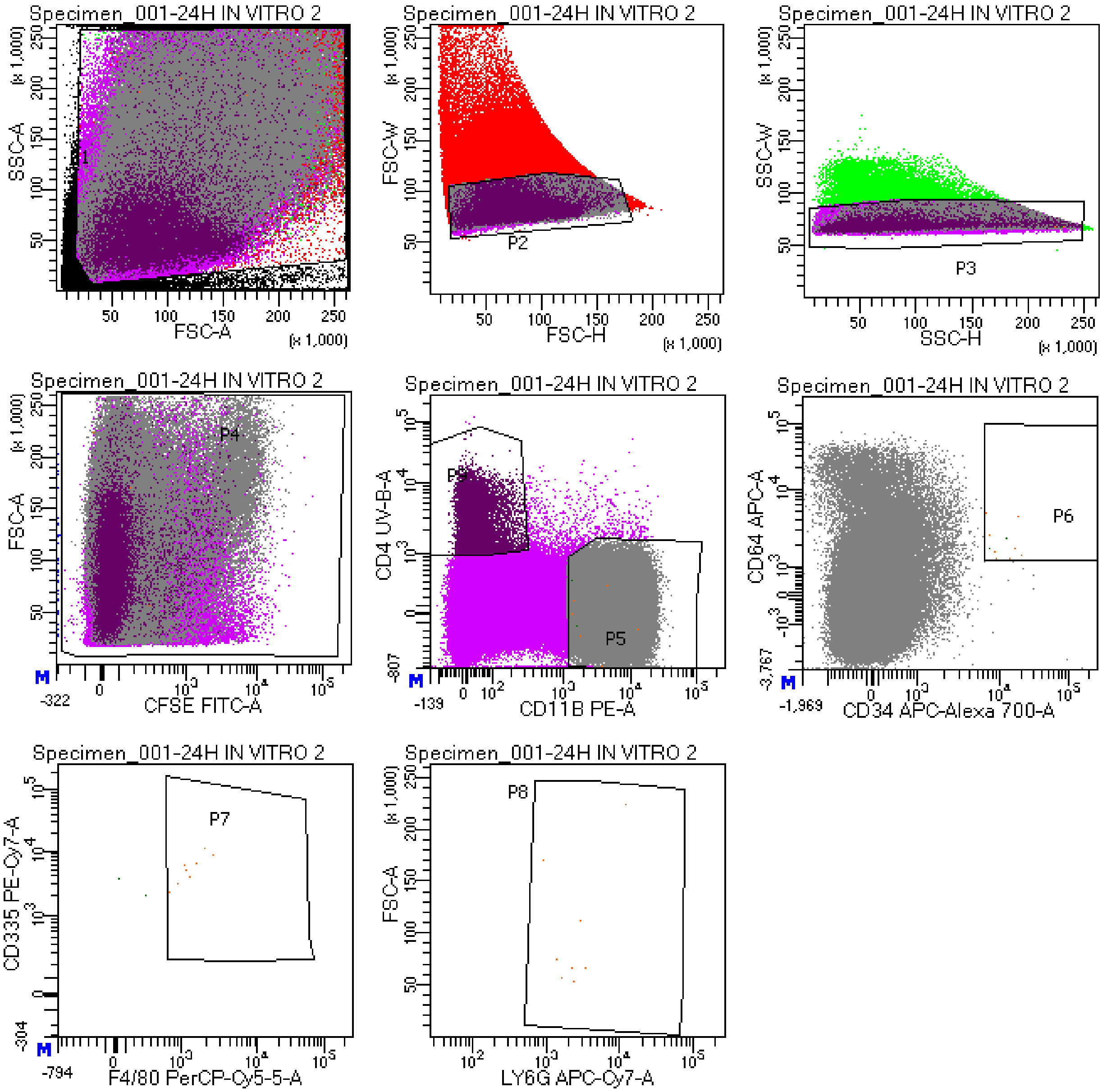

Tube: 24H IN VITRO 2

Population	#Events	%Parent	%Total
All Events	2,000,000	####	100.0
P1	1,724,711	86.2	86.2
P2	1,656,589	96.1	82.8
P3	1,646,738	99.4	82.3
P4	1,646,697	100.0	82.3
P5	295,948	18.0	14.8
P6	10	0.0	0.0
P7	8	80.0	0.0
P8	8	100.0	0.0
P9	11,682	0.7	0.6

BD FACSDiva 9.0.1

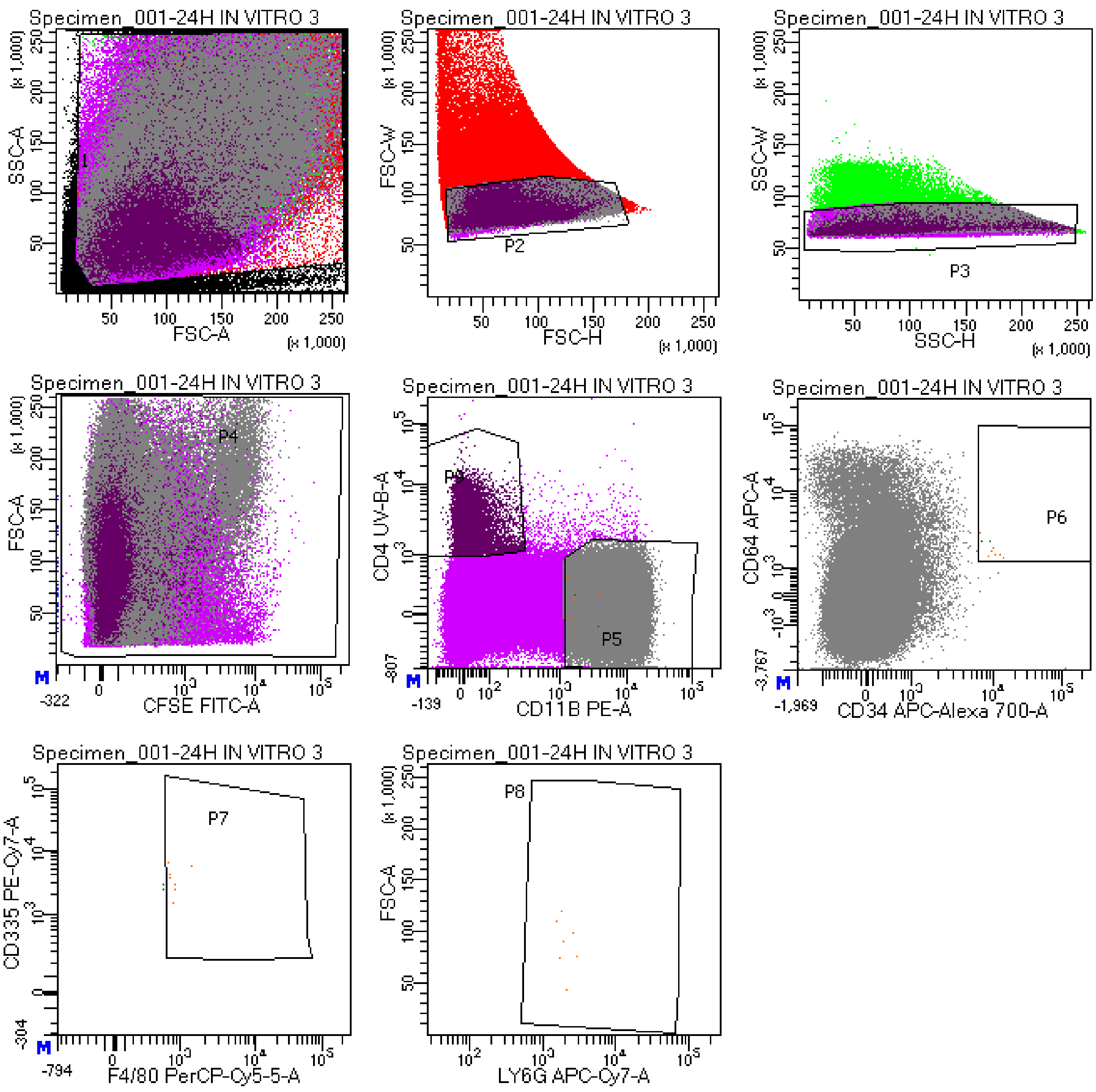

Tube: 24H IN VITRO 3

Population	#Events	%Parent	%Total
All Events	2,000,000	####	100.0
P1	1,749,226	87.5	87.5
P2	1,668,166	95.4	83.4
P3	1,657,267	99.3	82.9
P4	1,657,228	100.0	82.9
P5	266,639	16.1	13.3
P6	9	0.0	0.0
P7	7	77.8	0.0
P8	7	100.0	0.0
P9	10,972	0.7	0.5

BD FACSDiva 9.0.1

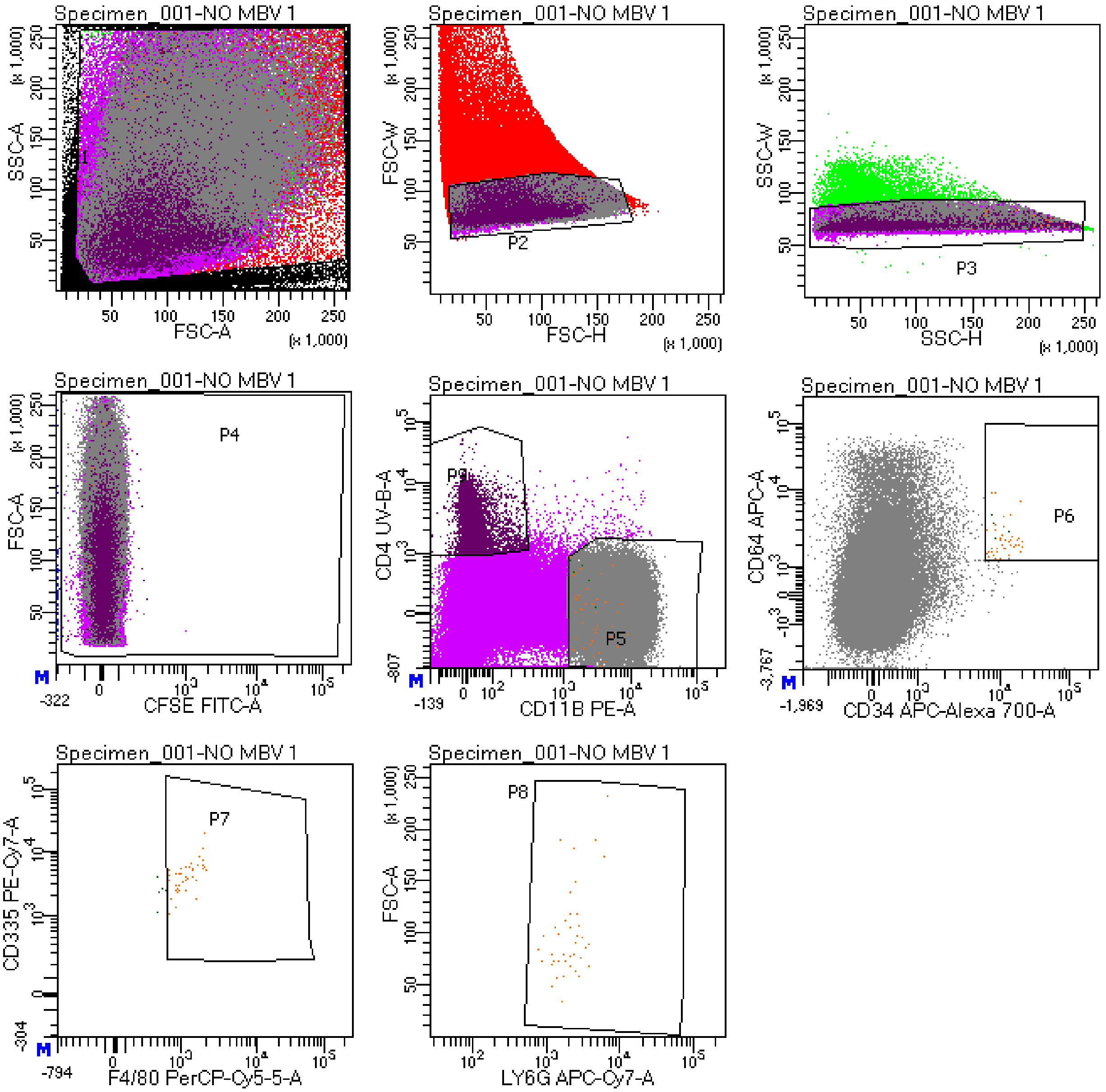

Tube: NO MBV 1

Population	#Events	%Parent	%Total
All Events	1,271,599	####	100.0
P1	1,067,539	84.0	84.0
P2	1,013,748	95.0	79.7
P3	1,009,026	99.5	79.4
P4	1,008,977	100.0	79.3
P5	172,740	17.1	13.6
P6	44	0.0	0.0
P7	39	88.6	0.0
P8	39	100.0	0.0
P9	6,197	0.6	0.5

BD FACSDiva 9.0.1

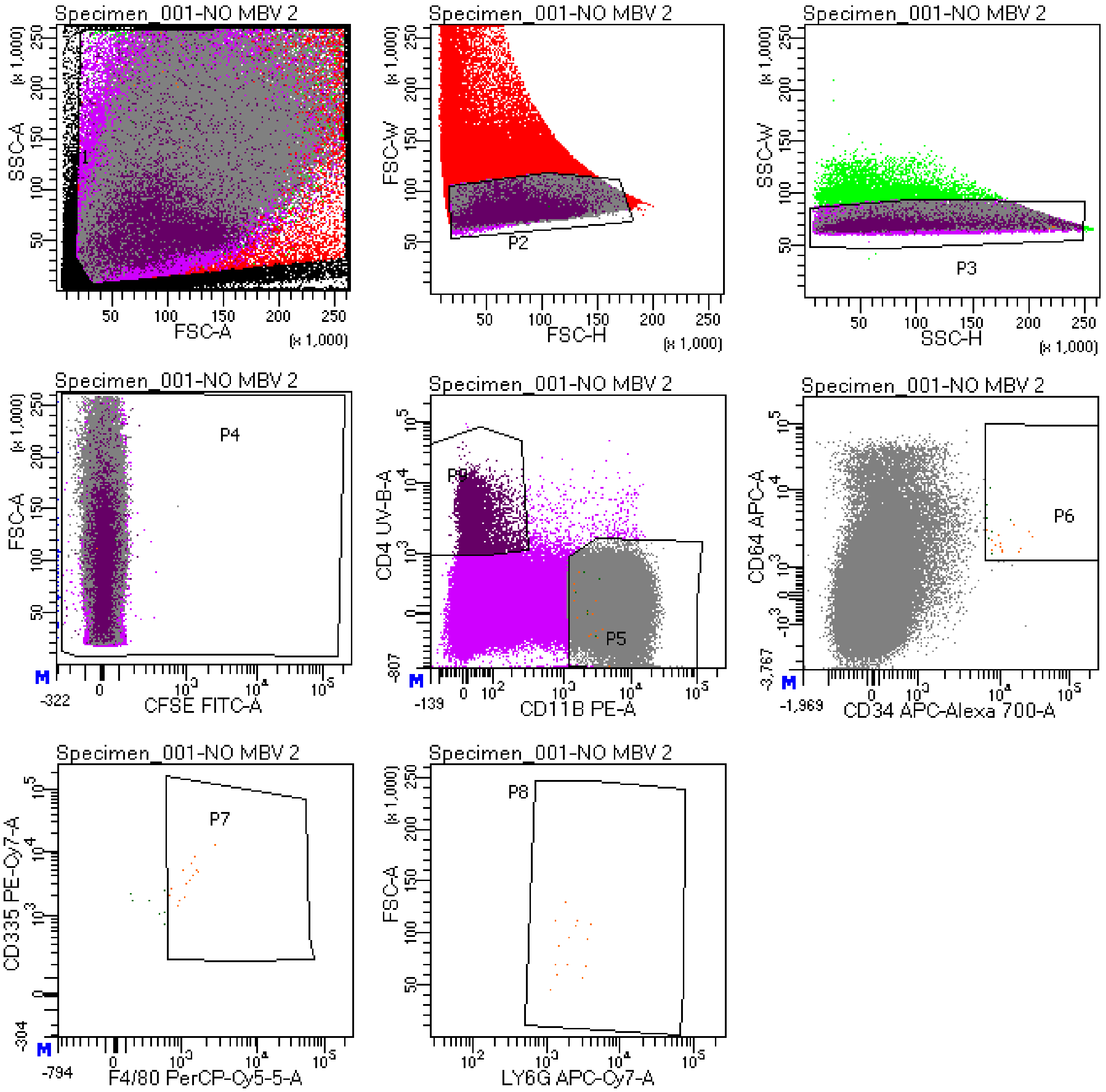

Tube: NO MBV 2

Population	#Events	%Parent	%Total
All Events	1,338,695	####	100.0
P1	1,102,928	82.4	82.4
P2	1,060,254	96.1	79.2
P3	1,053,612	99.4	78.7
P4	1,053,569	100.0	78.7
P5	228,587	21.7	17.1
P6	21	0.0	0.0
P7	14	66.7	0.0
P8	14	100.0	0.0
P9	7,400	0.7	0.6

BD FACSDiva 9.0.1

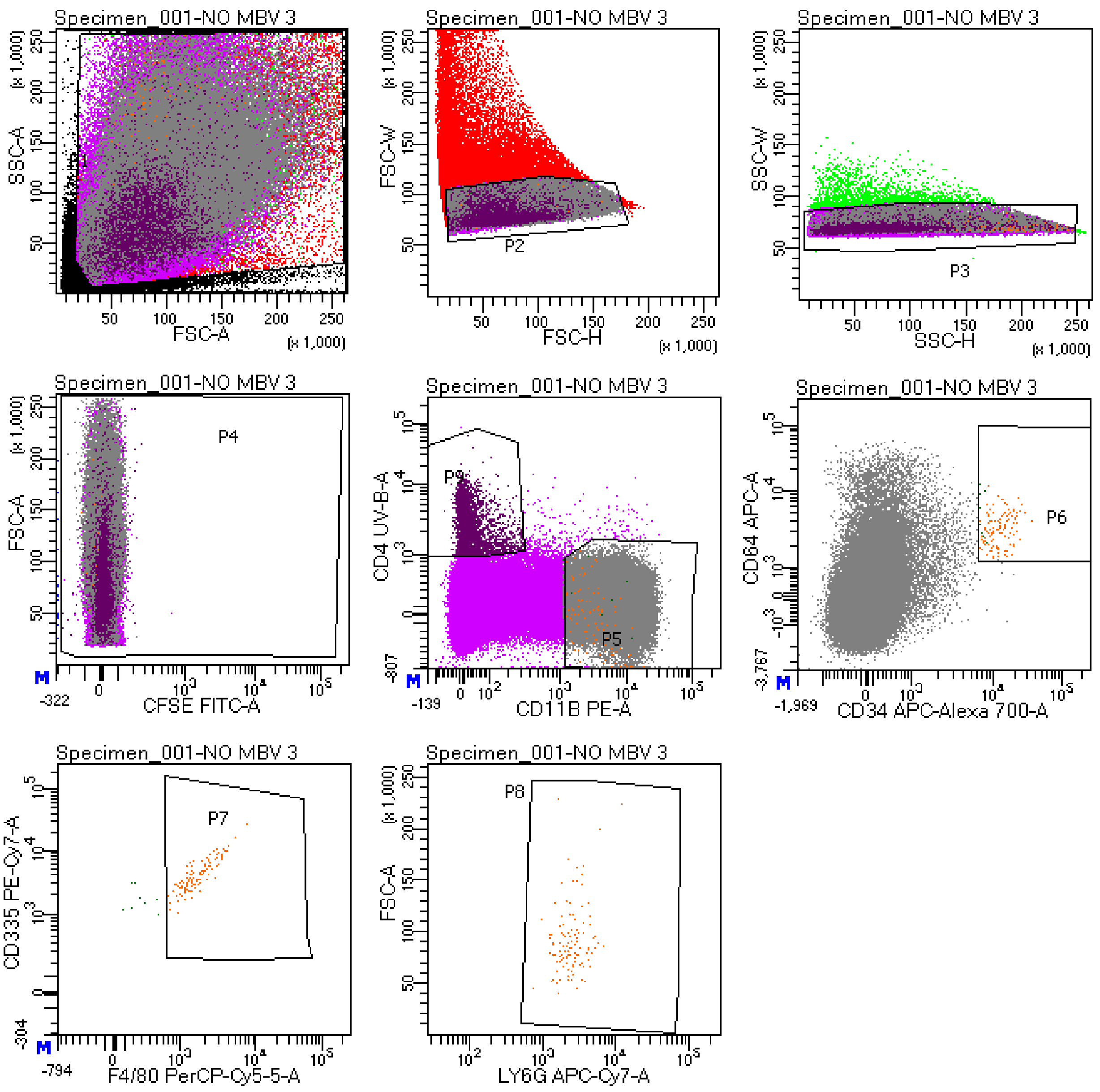

Tube: NO MBV 3

Population	#Events	%Parent	%Total
All Events	809,494	####	100.0
P1	668,782	82.6	82.6
P2	645,328	96.5	79.7
P3	642,659	99.6	79.4
P4	642,640	100.0	79.4
P5	124,096	19.3	15.3
P6	118	0.1	0.0
P7	110	93.2	0.0
P8	110	100.0	0.0
P9	3,952	0.6	0.5

BD FACSDiva 9.0.1

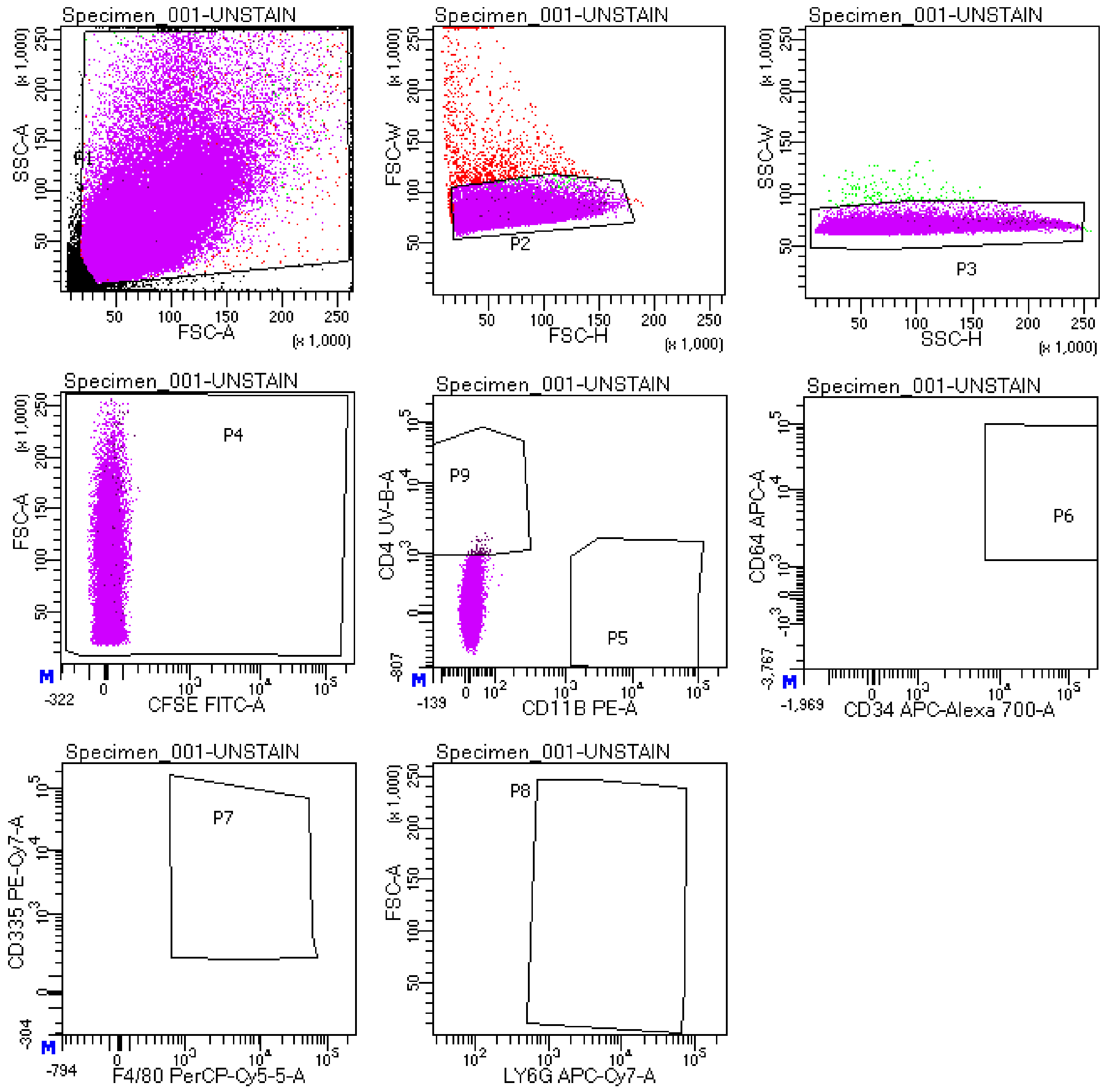

Tube: UNSTAIN

Population	#Events	%Parent	%Total
All Events	100,000	####	100.0
P1	68,985	69.0	69.0
P2	67,638	98.0	67.6
P3	67,477	99.8	67.5
P4	67,477	100.0	67.5
P5	0	0.0	0.0
P6	0	####	0.0
P7	0	####	0.0
P8	0	####	0.0
P9	67	0.1	0.1
